# Large-scale RNA-binding protein tethering screen discovers modulators of circular RNA metabolism

**DOI:** 10.1038/s41594-026-01911-7

**Published:** 2026-11-04

**Authors:** Tao Yu, Hsuan-Lin Her, Katrina Hou, Katherine L. Rothamel, Kevin Rhine, Brian A. Yee, Lijuan Kan, Qishan Liang, Shuhao Xu, Demetrius Tang, Caitlin Choi, Lena A. Street, Jack Naritomi, Grady Nguyen, Orel Mizrahi, Ester J. Kwon, Marko Jovanovic, Eric C. Lai, Gene W. Yeo

**Affiliations:** 1 Department of Cellular and Molecular Medicine, University of California San Diego, La Jolla, CA USA; 2 Sanford Stem Cell Institute and Stem Cell Program, University of California San Diego, La Jolla, CA USA; 3 Institute for Genomic Medicine, University of California San Diego, La Jolla, CA USA; 4 Center for RNA Technologies and Therapeutics, University of California San Diego, La Jolla, CA USA; 5 Developmental Biology Program, Sloan Kettering Institute, New York, NY USA; 6 Department of Biological Sciences, Columbia University, New York, NY USA; 7 Department of Bioengineering, University of California San Diego, La Jolla, CA USA; 8 Sanford Laboratories for Innovative Medicines, La Jolla, CA USA

**Keywords:** Non-coding RNAs, High-throughput screening

## Abstract

Circular RNAs (circRNAs) are emerging as important regulators in mammalian cells and promising substrates for RNA therapeutics, yet the *trans*-acting factors governing their metabolism remain poorly defined. Here we systematically evaluate 730 RNA-binding proteins (RBPs) using a tethered reporter assay and identify 281 RBPs that significantly modulate circRNA activity. Follow-up assays implicate these RBPs across key stages of the circRNA life cycle, including biogenesis, nuclear export, degradation and translation. We identify seven RBPs for tethered translation enhancement of therapeutically applicable circRNA vectors and highlight LARP4B as a potent enhancer of exogenous circRNA translation across multiple internal ribosomal entry sites and cell types. We uncover an evolutionarily conserved role for RBM15 in circRNA biogenesis in fly, mouse and human, further demonstrating a role in circRNA nuclear retention, and that perturbing RBM15 sites with dCasRx or antisense oligonucleotides modulates endogenous circRNA expression. Together, our study provides a global view of circRNA *trans*-regulation and describes practical entry points for therapeutic development.

## Abstract (web summary)

This study systematically evaluates proteins that control circular RNA production, stability and translation, revealing new regulators and strategies to enhance or suppress circular RNA activity for therapeutic development.

## Main

Circular RNAs (circRNAs) are covalently closed RNA species predominantly generated in the nucleus by backsplicing, where a downstream 5′ splice site is joined to an upstream 3′ splice site to form a loop^1,2^. Once exported, circRNAs can function as microRNA sponges, protein decoys and translation templates^3,4^, and are degraded via microRNA-mediated^5^, RNase P-mediated^6^ and virus-induced RNase L-mediated^7^ pathways, with others still undefined^8^. circRNAs are also gaining attention as vaccine and therapeutic vectors owing to their increased stability and reduced immunogenicity compared with mRNAs^9,10^. Translation efficiency remains a notable challenge, as current antigen-expressing RNA constructs often exhibit suboptimal protein output^11,12^. Enhancing translation efficiency could reduce delivery dose, thereby substantially lowering manufacturing costs^13^. Previous luciferase- and fluorescence-based reporters have characterized *cis*-acting sequence elements that enhance translation^9,14^, but offer limited insight into the RNA-binding proteins (RBPs) recruited to these elements.

The identities and roles of RBPs in circRNA biogenesis and metabolism remain largely unaddressed, limiting both our understanding of circRNA regulation and our ability to engineer highly efficacious therapeutic vectors. Only a handful of RBPs (for example, QKI, FUS, MBNL1 and SFPQ) are known to bind the intronic regions flanking circularizing exons^15–20^. However, given the low conservation of non-coding intronic sequences across species, the existence of a conserved *trans*-regulatory mechanism remains uncertain.

Here we developed tethered luciferase-based circRNA reporter assays to systematically test RBPs as circRNA effectors, screening 730 human RBPs (846 isoforms), identifying 281 RBPs (296 isoforms) as candidate effectors and proposing regulatory modalities throughout the circRNA life cycle. We further developed an exogenous circRNA reporter system and demonstrated that LARP4B broadly enhances circRNA translation across various internal ribosomal entry site (IRES) elements and cell lines. LARP4B prefers to bind AU-rich sequences, and incorporating poly(A) or poly(T) sequences enhances protein production of circRNA vectors. An additional focused screen extended this toolbox with six more RBPs that enhance exogenous circRNA translation across multiple cell lines. In parallel, we identified and demonstrated RBM15 as an unexpected multifaceted regulator of endogenous circRNAs, promoting circRNA biogenesis and retaining circRNAs in the nucleus. RBM15 directly binds exonic sequences near backsplicing junctions (BSJs) and is a conserved *trans*-regulatory factor that controls circRNA biogenesis in humans, mice and flies. Blocking the binding sites of RBM15 with dCasRx or antisense oligonucleotides (ASOs) is sufficient to modulate endogenous circRNA expression, unlocking the limitation of previous methods that only targeted the BSJs. Together, these findings uncover unexpected *trans*-acting regulators of circRNAs and inform therapeutic development by revealing translation-enhancing strategies and alternative approaches for suppressing endogenous circRNA expression.

## Results

### Establishment of tethered circRNA reporter system

Previous RBP-regulator screens relied on reporters with pre-existing *cis*-regulatory elements in flanking introns^18,20^, limiting evaluation of RBPs with uncharacterized preferences and identification of exonic regulators. A recent CRISPR screen utilized a single-exon derived circRNA reporter and identified ZC3H14 as a regulator binding the exon–intron boundary^21^, but still depended on reporter sequence context. We reasoned that forcibly tethering RBPs to a circRNA reporter would allow more comprehensive evaluation of the regulatory potential of RBPs.

**Fig. 1:**
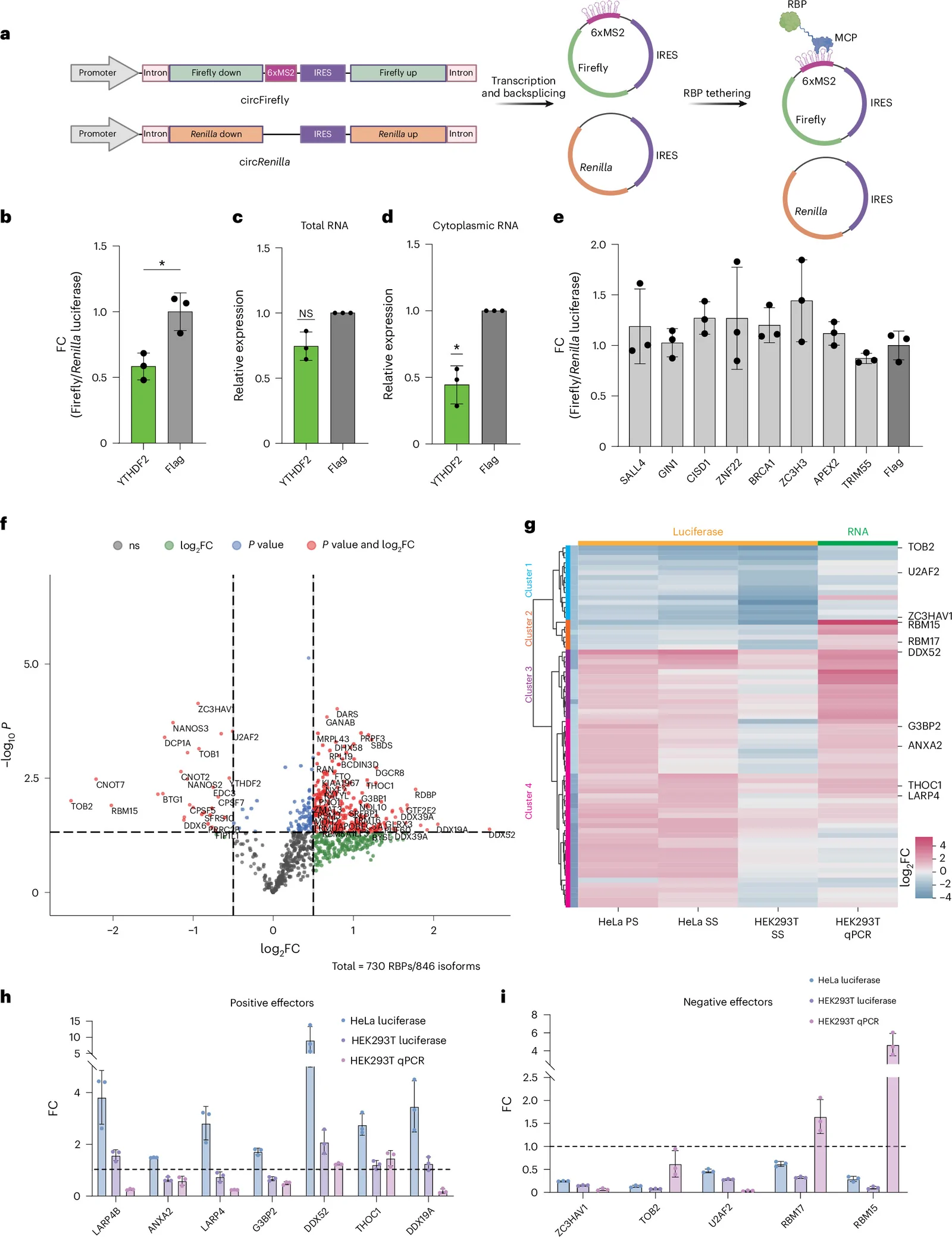
A circRNA luciferase reporter system for RBP tethering screen. **a**, Schematic design of the circFirefly and circ*Renilla* reporters. Upon backsplicing, split Firefly and *Renilla* fragments form intact luciferase genes and are translated, driven by encephalomyocarditis virus IRES. circFirefly reporter carries 6x MS2 sequence for RBP tethering through MCP fusions. **b**, Luciferase assays of the positive control (mean ± s.d. of biological replicates, *n* = 3). Luciferase signals were normalized to the Flag control. Welch’s *t*-test, two-tailed. *P = 0.018. **c**, qPCR of circFirefly RNA in total RNA extracts (mean ± s.d. of biological replicates, *n* = 3). Relative expression was normalized to the Flag control. One-sample *t*-test, two-tailed. NS (not significant), *P* = 0.0562. **d**, qPCR of circFirefly RNA in cytoplasmic RNA fractions (mean ± s.d. of biological replicates, *n* = 3). Relative expression was normalized to the Flag control. One-sample *t*-test, two-tailed. *P = 0.0213. **e**, Luciferase assays of the negative controls (mean ± s.d. of biological replicates, *n* = 3). Luciferase signals were normalized to the Flag control. **f**, Volcano plot of RBP tethering screen results. Cutoff is set at |log_2_FC| > 0.5 and FDR < 0.05. Red dots represent RBP candidates that pass both cutoffs. Blue and green dots represent RBPs that only pass one of the cutoffs. Gray dots represent RBPs that do not pass either cutoff. Multiple *t*-test, two-tailed with FDR correction. **g**, Heatmap summary of secondary screen results. *K*-means clustered heatmap shows the log_2_FC of luciferase assays in two cell lines and qPCR results from HEK293T cells. PS, primary screen; SS, secondary screen. **h**,**i**, Bar plots showing examples of RBPs from different clusters (mean ± s.d. of biological replicates, *n* = 3). The first two bars in each group present luciferase results from the secondary screen. The third bar in each group presents qPCR results from separate experiments with biological replicates. FCs are all normalized to the corresponding Flag controls. Panel **a** created in BioRender; Yu, T. https://biorender.com/56v8wjl (2026). Source data

Therefore, we designed two luciferase minigene reporters containing minimal *ZKSCAN1*^22^ intronic sequences that facilitate backsplicing, splitting the Firefly or *Renilla* luciferase gene into two fragments. Intact Firefly or *Renilla* luciferase is only produced upon successful backsplicing. Translation was initiated using the encephalomyocarditis virus IRES. The Firefly reporter (circFirefly) contained 6x MS2 hairpins to recruit MS2 coat protein (MCP)-RBP fusions, with the *Renilla* reporter (circ*Renilla*), lacking MS2 hairpins, as an internal control (Fig. 1a and Methods). We used a circular rather than a linear RNA control to avoid excluding RBPs affecting both circRNAs and their linear counterparts.

**Extended Data Fig. 1:**
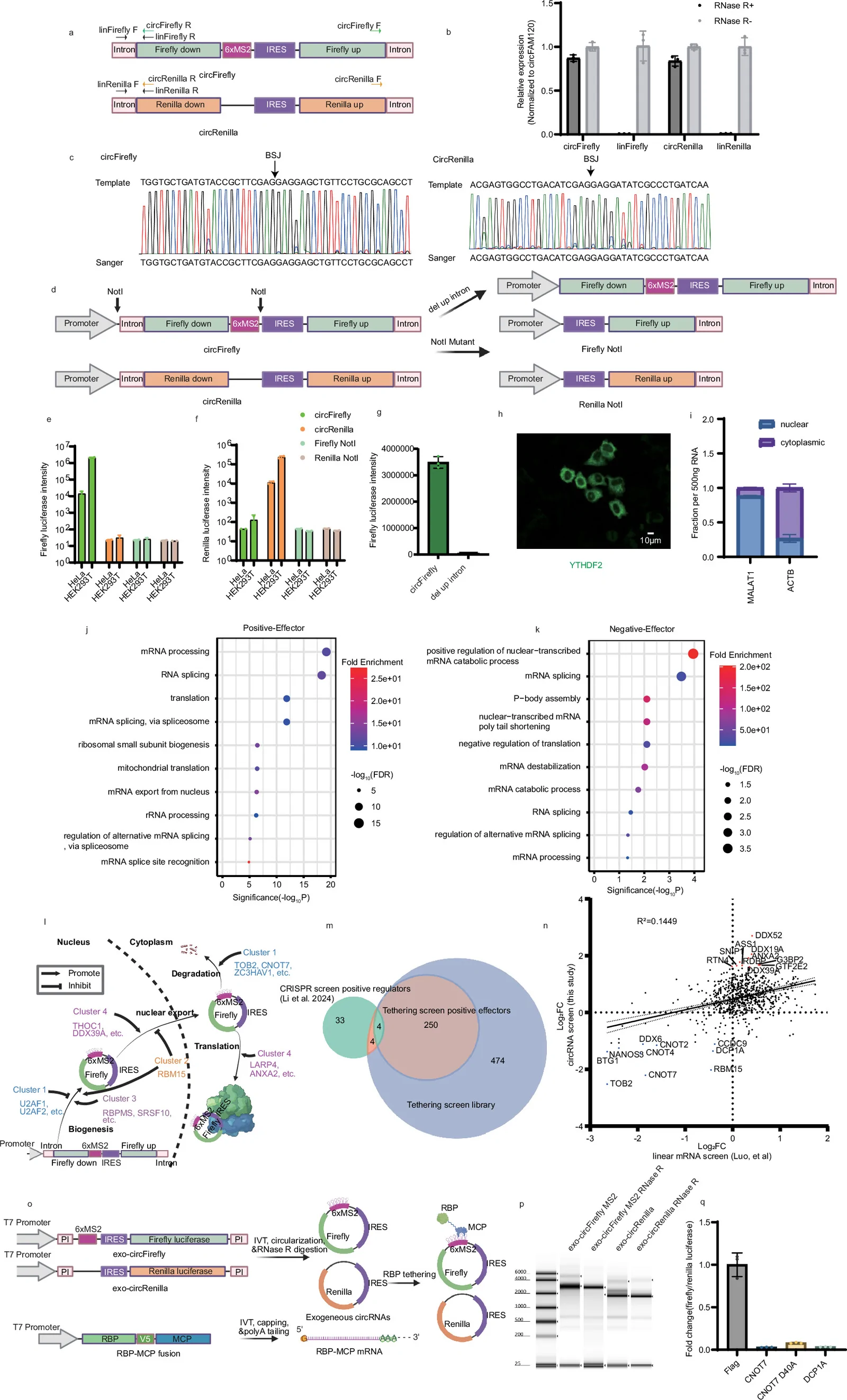
Validation of circRNA luciferase reporter system and tethering screen candidates. **a**. Schematic view of divergent and convergent primer design. **b**. Bar plot showing the RNA level comparison of circFirefly, circRenilla, linFirefly and linRenilla before and after RNase R treatment by qPCR (mean ± s.d., n = 3). Error bars represent standard deviations of biological replicates. **c**. Sanger sequencing of the qPCR product detected successful backsplicing junctions. **d**. Schematic view of truncated circFirefly and circRenilla mutants generated. The circularization deficient mutants are used to confirm that the upstream fragments do not generate luciferase signals. **e**, **f**. Bar plots showing raw Firefly and Renilla luciferase signals of circFirefly and circRenilla reporters and their mutants in Hela and HEK293T cells (mean ± s.d., n = 2). Error bars represent standard deviations of biological replicates. **g**. Bar plot showing raw Firefly luciferase signals, demonstrating del up intron mutant (that prevent backsplicing) does not generate complementary protein to produce luciferase signal (mean ± s.d., n = 3). Error bars represent standard deviations of biological replicates. **h**. Immunofluorescence staining of YTHDF2-MCP and METTL3-MCP RBP fusions. Green: V5 stained fusion proteins. **i**. Stacked bar plot showing the performance of cell fractionation (mean ± s.d., n = 3). Samples are normalized by RNA loading of 500 ng. Error bars represent standard deviations of biological replicates. **j**. Gene Ontology analysis of positive effectors. Background list is set to all RBPs in the tethering library. Top 10 GO terms in Biological Processes are shown. **k**. Gene Ontology analysis of negative effectors. Background list is set to all RBPs in the tethering library. Top 10 GO terms in Biological Processes are shown. **l**. Proposed mechanisms of different clusters. Cluster 1 is hypothesized to inhibit biogenesis or promote degradation. Cluster 2 is hypothesized to play multimodal functions, for example both promoting biogenesis and inhibiting nuclear export. Cluster 3 is hypothesized to promote biogenesis, stability and/or translation. Cluster 4 is hypothesized to promote nuclear export or translation. Created with Biorender.com **m**. Venn diagram showing overlap between our tethering screen and a recent CRISPR screen to identify RBP regulators of circRNA biogenesis. **n**. Scatter plot comparison between circRNA tethering screen with mRNA tethering screen. Solid line presents the linear regression. Top ten upregulators in circRNA screen are highlighted in red and top ten downregulators in circRNA screen are highlighted in blue. **o**. Schematic design of exogenous circRNA luciferase reporter system. exo-circFirefly and exo-circRenilla circRNAs can be produced through in vitro transcription, circularization with the permuted intron and purification through RNase R digestion. RBP-MCP mRNAs can be made by in vitro transcription with capping and tailing. All three components are delivered together to assess the effect of an RBP on exogenously delivered circRNAs. Created with Biorender.com **p**. Tapestation result of exo-circFirefly and exo-circRenilla RNA reporters showing the purity of circRNAs after RNase R digestion. **q**. Luciferase assay of tethering CNOT7 and mutant CNOT7 D40A and DCP1A to the exo-circFirefly reporter system (mean ± s.d., n = 3). Error bars represent standard deviations of biological replicates. a,d,l,o are created with Biorender.com. Yu, T. (2026) https://BioRender.com/b4rwfaj. Source data

We confirmed successful circularization and ruled out *trans*-splicing by RNase R treatment and quantitative PCR (qPCR) with divergent and convergent primers: only circRNA products resisted RNase R, and we confirmed the BSJs by Sanger sequencing (Extended Data Fig. 1a–c). To exclude truncated-protein artifacts, we generated mutants producing only the Firefly-up/*Renilla*-up fragments (Firefly NotI and *Renilla* NotI) or lacking the upstream intron entirely (del up intron); only the intact circFirefly and circ*Renilla* constructs produced luminescence in HeLa and HEK293T cells, while all mutants produced almost no signal (Extended Data Fig. 1d–g). These results indicated that the reporter specifically reflected translation of circRNA-derived luciferase.

To validate assay sensitivity, we tethered YTHDF2, a known circRNA-degrading RBP^6^, via MCP fusion (Flag–MCP as control). YTHDF2 tethering decreased luciferase signal by 40% (Fig. 1b), and reduced circFirefly RNA levels, particularly in the cytoplasmic fraction, consistent with its predominantly cytoplasmic localization (Fig. 1c,d and Extended Data Fig. 1h,i). Eight zinc-finger-containing transcription factors with minimal RNA binding/regulation^23^ did not alter circRNA expression when tethered, suggesting relatively low false discovery rate (FDR) (Fig. 1e). Together, these results demonstrate that the reporter system reliably reflects the functional effect of RBPs on circRNAs.

### circRNA tethering screen proposes RBP functions throughout circRNA life cycle

Using our previously developed RBP tethering library, we systematically evaluated how 730 RBPs (846 unique isoforms), fused to MCP, affect circRNA expression^24,25^ via arrayed luciferase assay in triplicate in HeLa cells, with Flag–MCP controls on each plate for normalization. With a cutoff of |log_2_(fold change (FC))| > 0.5 and FDR < 0.05, we identified 296 candidate RBP isoforms (281 RBPs), including 267 positive and 29 negative effectors of relative luciferase signal (Fig. 1f). Notably, YTHDF2 was retrieved, underscoring assay sensitivity and reproducibility (Supplementary Table 1).

Gene Ontology (GO) analysis (using the RBP library as background) showed positive effectors enriched for RNA splicing, transport and translation factors, and negative effectors enriched for RNA degradation and splicing factors (Extended Data Fig. 1j,k). Positive effectors included translation initiation factors and ribosomal subunits that might promote translation, splicing factors that might enhance biogenesis, and both protein (RAN and XPO1) and mRNA (NXF2, THOCs and DDX39A/B) nuclear export factors, consistent with both pathways contributing to circRNA export^26,27^. Negative effectors included ZC3HAV1 (ZAP), a circRNA-degrading factor in exogenous RNA surveillance^28^, and, surprisingly, mRNA decapping/de-polyadenylation factors (DCP1A, TOB2 and CNOT2/4/7) despite circRNAs lacking a 5′ cap or poly(A) tail. Notably, U2AF1 and U2AF2 emerged as negative effectors, likely reflecting position-dependent binding, per our prior splicing regulator screen^24^, underscoring a limitation of tethering assays for position-sensitive RBPs (Supplementary Table 1).

To increase confidence and probe mechanism, we tested top candidates across GO terms in a secondary screen in HeLa and HEK293T cells with RNA-level (qPCR) measurements (Supplementary Table 2). Results were highly reproducible in HeLa cells; in HEK293T cells, negative effectors were similarly consistent, but the positive effectors exhibited more cell-type specificity. Integrating the luciferase and RNA-level data across both lines, hierarchical clustering revealed four clusters (Fig. 1g), with selected candidates validated by qPCR (Fig. 1h,i). Cluster 1 (decreased RNA and luciferase levels) included likely degradation factors (for example, ZC3HAV1^29^, TOB2 and DCP1A^30^) and biogenesis inhibitors (for example, U2AF2^31^) (Fig. 1g,i). Cluster 2 (increased RNA and decreased luciferase levels) indicated multifunctional regulators (for example, RBM15, below) (Fig. 1g,i). Cluster 3 (increased RNA and luciferase levels) suggested enhancers of biogenesis, stability or translation (Fig. 1g,h). Cluster 4 (modest RNA change and increased luciferase) included nuclear export (for example, THOC1^32^) or translation-promoting (for example, LARP4^33^) factors (Fig. 1g,h). Several uncharacterized candidates (for example, RBM17 and DDX52) also emerged; hypothesized mechanisms for these RBPs are summarized in Extended Data Fig. 1l and provide a framework for future mechanistic studies.

Of the 41 positive regulators from the aforementioned CRISPR screen^21^, 8 were included in our tethering library, and 4 were identified as positive effectors, including their top candidate ZC3H14 (Extended Data Fig. 1m), highlighting our screen’s reliability and the sensitivity for exonic circRNA regulators. Comparing with our previous tethering screen for mRNA stability and translation effectors^25^, we observed a modest correlation (*R*^2^ = 0.1449). Stronger concordance between the two screens was observed among the top negative effectors than top positive effectors, suggesting that degradation pathways are more shared, while biogenesis and translation effectors are more circRNA-specific (Extended Data Fig. 1n).

Given that decapping and deadenylation complexes consistently reduced luciferase signal in two cell lines, we tested whether this reflects action on linear precursors before backsplicing or direct degradation of circRNAs that may be cap/poly(A)-independent, using redesigned luciferase reporters (exo-circFirefly and exo-circ*Renilla*) that generate in vitro-transcribed circRNAs by group I introns (Extended Data Fig. 1o). With in vitro-synthesized circRNA enriched by RNase R digestion (Extended Data Fig. 1p), RBP-MCP fusion genes were co-delivered as in vitro-transcribed mRNAs. Interestingly, tethering CNOT7 and DCP1A to the in vitro-synthesized circRNA reporter still decreased the luciferase signal by over 90%, ruling out action on linear precursors. An enzymatically dead CNOT7 mutant (D40A)^34^ also decreased signal (Extended Data Fig. 1q), while DCP2, the catalytic subunit in the decapping complex, did not (Supplementary Table 1), suggesting independent functions of their known enzymatic activities, which merits future investigation.

Together, our screen identifies both known and unexpected circRNA effector RBPs and infers distinct classes of regulators across the circRNA life cycle.

### Identification of RBPs promoting circRNA translation

**Extended Data Fig. 2:**
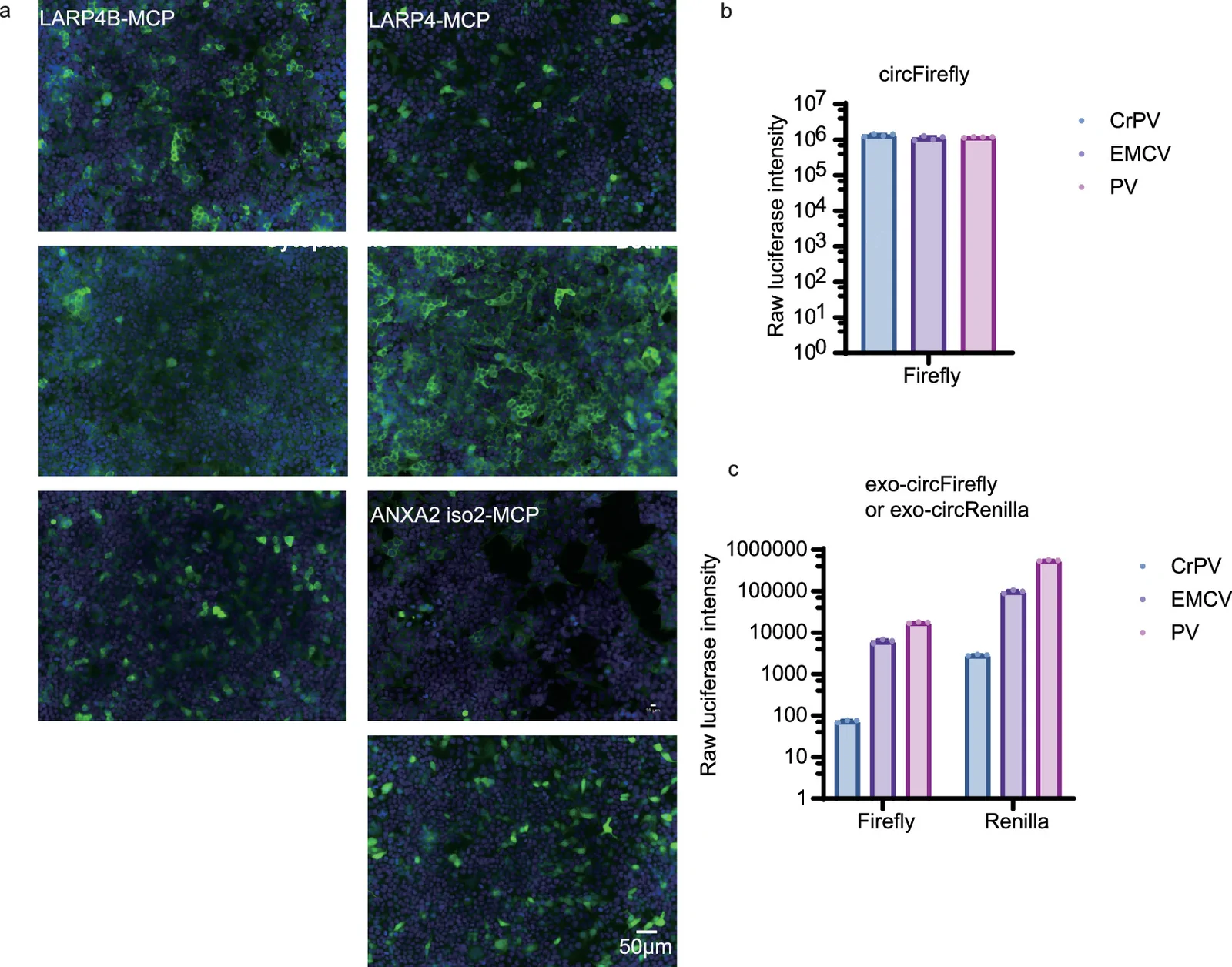
Identification of translation promoting RBPs. **a**. Immunofluorescence of RBP-MCP fusion proteins translated from the in vitro transcribed mRNAs. Green: V5 stained RBP-MCP fusion proteins; Blue: DAPI **b**. Raw luciferase signal of different IRES driving circFirefly reporter (by backsplicing in the cells) (mean ± s.d., n = 4). Error bars represent standard deviations of biological replicates. **c**. Raw luciferase signal of different IRES driving exo-circFirefly or exo-circRenilla reporters (by in vitro circularization with permuted introns) (mean ± s.d., n = 3). Error bars represent standard deviations of biological replicates. Source data

We next focused on two goals: identifying candidates that enhance translation of exogenous circRNAs for therapeutic vector design, and uncovering regulators of endogenous circRNAs. Reasoning that translation-promoting RBPs would be cytoplasmic, we selected six top candidate effectors with immunofluorescence-confirmed cytoplasmic localization (Supplementary Table 2 and Extended Data Fig. 2a) and tested them with the exo-circFirefly/exo-circ*Renilla* reporter system, which models exogenous circRNA delivery while eliminating confounding effects on splicing or nuclear export.

**Fig. 2:**
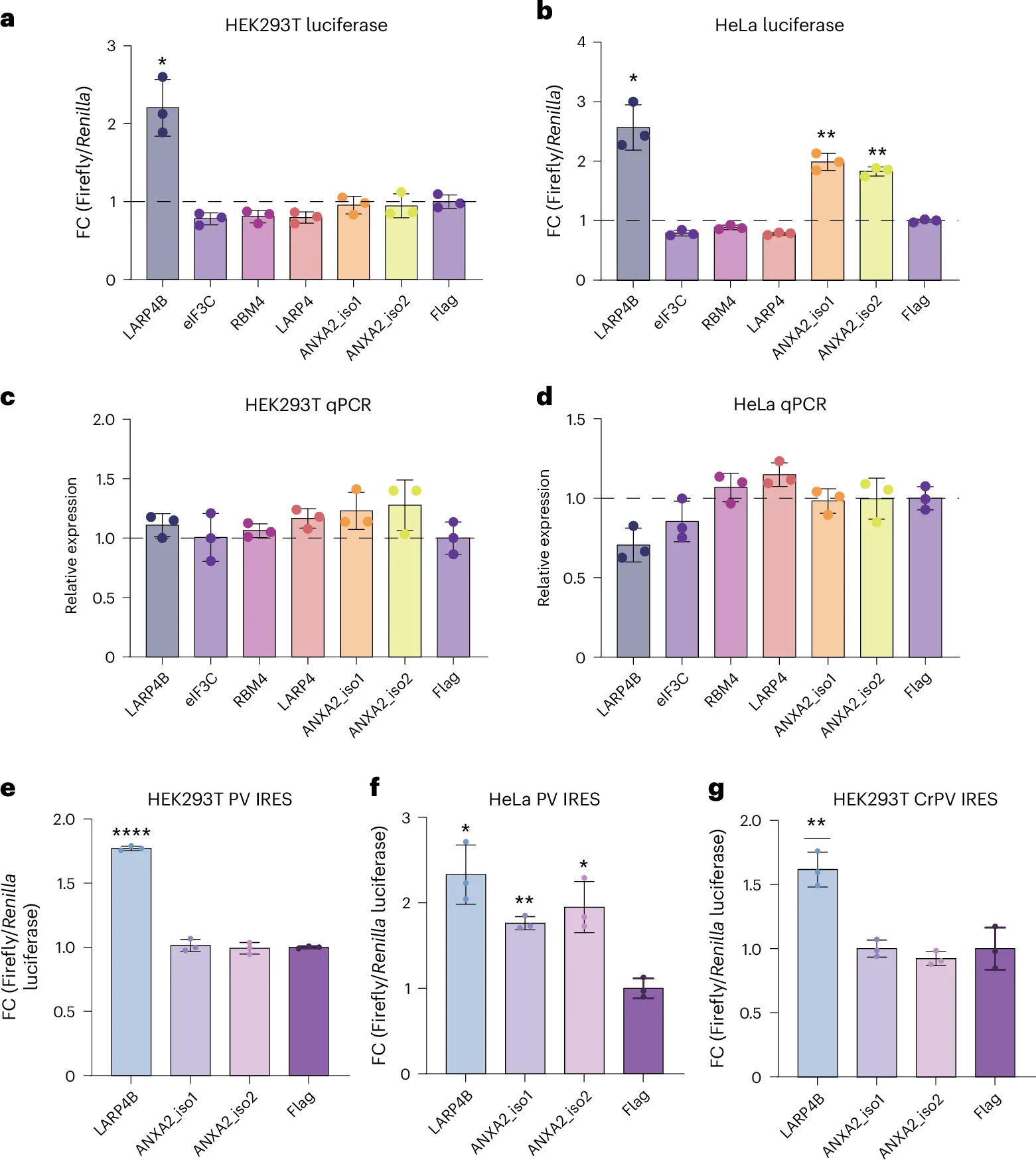
Identification of RBPs promoting circRNA translation. **a**,**b**, Luciferase assay showing effects of RBP tethering on protein production of exogenously delivered circRNAs in HEK293T (**a**) and HeLa (**b**) cells (mean ± s.d. of biological replicates, *n* = 3). Welch’s *t*-test, two-tailed. *P < 0.05, **P < 0.01. ANXA2_iso1: GYRBP004.H11; ANXA2_iso2: GYRBP004.A08. **c**,**d**, Relative RNA level by qPCR showing the effects of RBP tethering on circRNA stability of exogenously delivered circRNAs in HEK293T (**c**) and HeLa (**d**) cells (mean ± s.d. of biological replicates, *n* = 3). **e**,**f**, Luciferase assay showing effects of RBP tethering on protein production of exogenously delivered PV IRES-driven circRNAs in HEK293T (**e**) and HeLa (**f**) cells (mean ± s.d. of biological replicates, *n* = 3). Welch’s *t*-test, two-tailed. *P < 0.05, **P < 0.01, ****P < 0.0001. **g**, Luciferase assay showing effects of RBP tethering on protein production of exogenously delivered CrPV IRES-driven circRNAs in HEK293T cells (mean ± s.d. of biological replicates, *n* = 3). Welch’s *t*-test, two-tailed. **P = 0.0081. Source data

LARP4B consistently increased the luciferase signal more than twofold in both HEK293T and HeLa cells, while two ANXA2 isoforms increased signal approximately twofold only in HeLa cells, indicating cell-type-specific regulation (Fig. 2a,b). None of the six RBPs increased exo-circFirefly RNA levels, pointing to enhanced translation rather than RNA stabilization (Fig. 2c,d). Notably, the other four RBPs tested only upregulated signal from endogenously produced circFirefly, suggesting reliance on early RNA-processing steps (Fig. 2a,b and Supplementary Table 1).

To assess generalizability, we added two mechanistically distinct IRES elements^35^, namely poliovirus (PV) and cricket paralysis virus (CrPV), to the reporters. Interestingly, endogenously expressed circFirefly constructs via backsplicing produced comparable luciferase signals with either IRES (Extended Data Fig. 2b), whereas the exo-circFirefly/exo-circ*Renilla* constructs increased stepwise as their structures were extended to scaffold more IRES *trans*-acting factors, consistent with previous reports^9^ (Extended Data Fig. 2c). These findings support that circRNA processing history predefines translational competency and validate exogenous circRNA systems for optimizing therapeutic constructs. Tethering LARP4B and ANXA2 across these IRES elements showed that LARP4B consistently enhanced translation regardless of IRES, while ANXA2 remained HeLa cell-specific (Fig. 2e–g; CrPV signal was too low in HeLa cells to assess).

In summary, LARP4B broadly enhances circRNA translation, whereas ANXA2 functions as a cell-type-specific enhancer. Our exogenous reporter system reveals key differences between backsplicing-derived circRNAs and in vitro-synthesized circRNAs, underscoring the value of synthesized-circRNA reporters for therapeutic screening.

### Exo-circFirefly screen for RBPs that promote the translation of in vitro-synthesized circRNAs

**Fig. 3:**
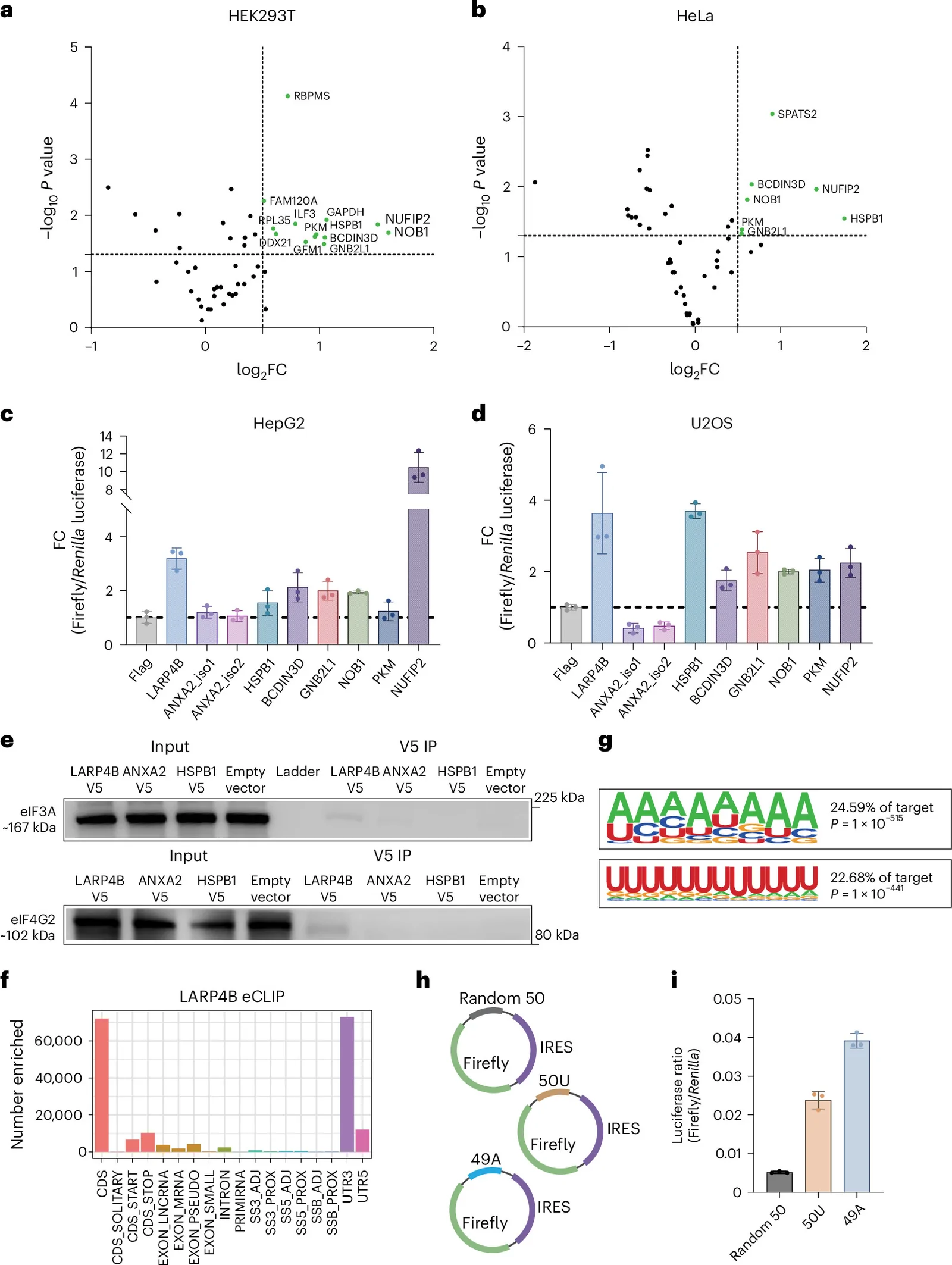
Expanding the toolbox of RBP tethering-mediated circRNA translation enhancement. **a**,**b**, Volcano plot showing the results of the focused screen for translation enhancers of exogenously delivered circRNA reporters in HEK293T **(a**) and HeLa (**b**) cells. Significant candidates (log_2_FC > 0.5 and *P* < 0.05) are highlighted green. Multiple *t*-test, two-tailed, *P* value not adjusted to allow more discoveries, which were validated later. **c**,**d**, Luciferase assay showing effects of RBP tethering on protein production of exogenously delivered circRNAs in HepG2 (**c**) and U2OS (**d**) cells (mean ± s.d. of biological replicates, *n* = 3) in HepG2 and U2OS cells. **e**, Western blot showing interaction of LARP4B with eIF3A and eIF4G2 with V5 pulldown. The interaction of LARP4B with eIF3A and eIF4G2 was repeated in two independent experiments. **f**, Bar plot showing the region distribution of LARP4B eCLIP binding sites. **g**, Selective top motifs of LARP4B binding sites showing poly(A) and poly(U) tracts. Adjusted *P* value from Homer motif analysis (based on Poisson distribution). **h**, Schematics showing the incorporation of 50U or 49A sequences into the circRNA vector to compare with random 50 sequence. **i**, Luciferase assay showing effects of 50U and 49A on translation enhancement of exogenously delivered circRNA reporters (mean ± s.d. of biological replicates, *n* = 3). Source data

To expand the translation-enhancing RBP toolbox, we selected 53 additional top candidates annotated as cytoplasmic in the Human Protein Atlas^36,37^, generated RBP-MCP mRNA for 50 and screened them in both HEK293T and HeLa cells with the exo-circFirefly reporter system. This identified 13 candidates in HEK293T cells and 7 in HeLa cells, with 6 overlapping (Fig. 3a,b and Supplementary Table 2), namely, NUFIP2, HSPB1, NOB1, BCDIN3D, PKM and GNB2L1 (Fig. 3a,b).

We further tested these six RBPs plus LARP4B and ANXA2 in HepG2 (liver) and U2OS (bone) cells. Most enhanced translation in both lines, while ANXA2 remained HeLa-specific; PKM retained activity in U2OS but not HepG2 cells, and NUFIP2 showed a particularly strong (around tenfold) effect in HepG2 cells (Fig. 3c,d). These results demonstrate generalizable but cell-type-dependent translation enhancement.

### LARP4B is associated with mRNA and circRNA translation

**Extended Data Fig. 3:**
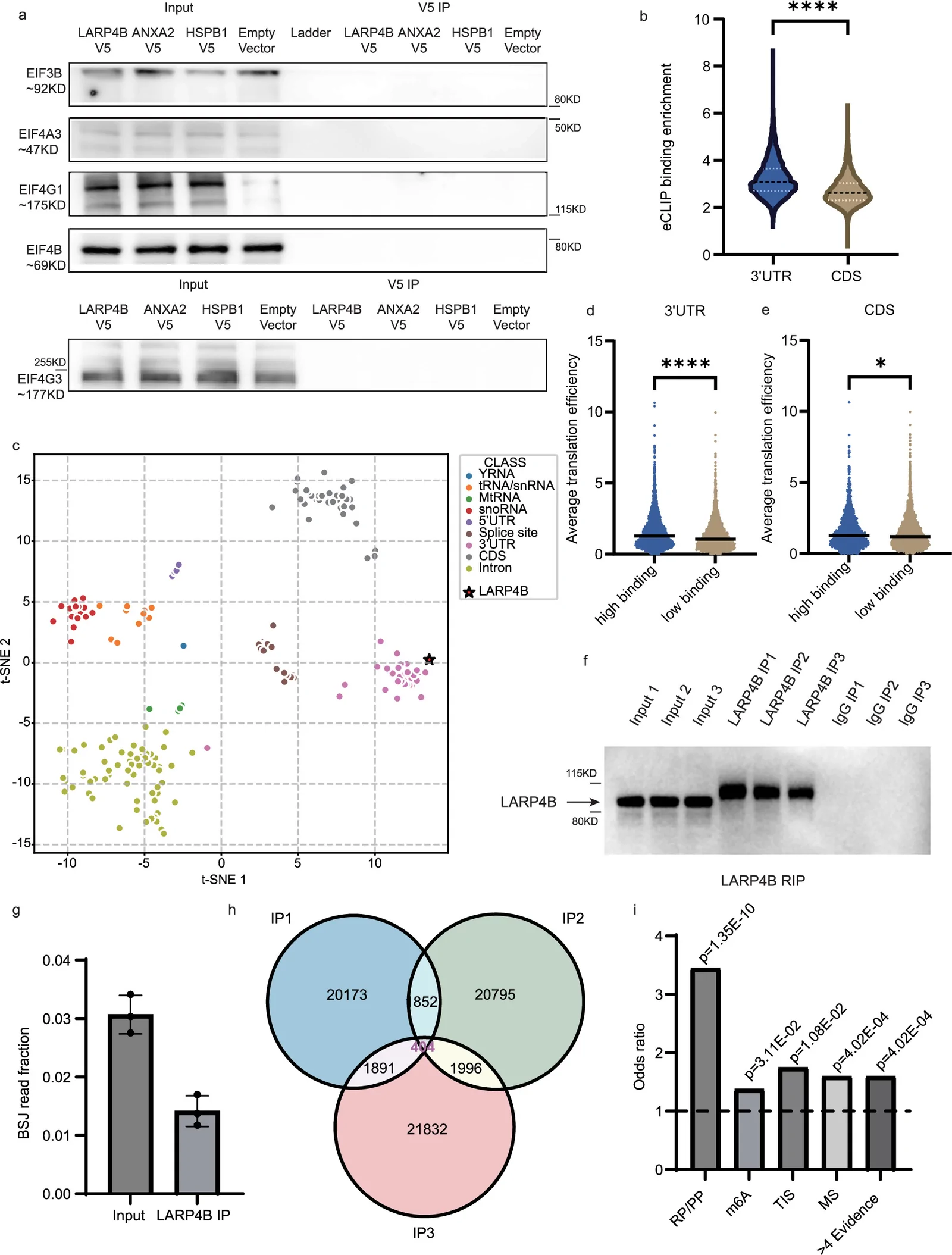
LARP4B eCLIP and RIP-circRNA-seq. **a**. Western blot showing no interaction between LARP4B with a number of other eIFs proteins tested. **b**. Violin plot showing LARP4B eCLIP binding level difference between 3′UTR and CDS. Black dash lines indicate the medians. Weich’s t-test, two-tailed. ****: p < 0.0001. **c**. t-SNE plot showing LARP4B eCLIP clustering close to 3′UTR binding RBPs from the ENCODE project. **d**. Translation efficiency analysis showing difference between transcript with high binding and low binding of LARP4B on 3′UTR. Weich’s t-test, two-tailed. ****: p < 0.0001. **e**. Translation efficiency analysis showing difference between transcript with high binding and low binding of LARP4B on CDS. Weich’s t-test, two-tailed. *: p < 0.05. **f**. Western blot showing successful pulldown of LARP4B for the RIP experiment. **g**. Bar plot showing factions of BSJ reads in input and LARP4B IP samples (mean ± s.d., n = 3). Error bars represent standard deviations of biological replicates. **h**. Venn diagram showing the overlap of significantly identified circRNAs between three biological replicates of LARP4B RIP experiments. Purple labels the number of reproducible circRNAs been identified. **i**. Enrichment of TransCirc evidence for reproducibly enriched circRNAs in LARP4B RIP. RP/PP: ribosome profiling/polysome profiling evidence; m6A: predicted m6A modification sites; TIS: predicted translation initiation sites; MS; mass spectrometry evidence (very few circRNAs have this evidence, might not be very suggestive). Source data

To probe the mechanism of LARP4B, we screened several eukaryotic translation initiation factors by co-immunoprecipitation (co-IP) and identified interactions between LARP4B and both eIF3A and eIF4G2. Neither factor was co-immunoprecipitated with ANXA2 or HSPB1, suggesting that these proteins might act through other mechanisms (Fig. 3e and Extended Data Fig. 3a). Notably, eIF4G2 has been implicated in IRES-dependent mRNA translation^38^ and *N*^6^-methyladenosine (m^6^A)-mediated circRNA translation^39^, potentially explaining the tethering effect of LARP4B and implicating broader relevance to m^6^A-mediated translation. The role of LARP4B in translation is also consistent with the reported association with the 40S ribosomal subunit and polysomes^40^.

Using enhanced crosslinking and immunoprecipitation (eCLIP) and RNA immunoprecipitation (RIP)–circRNA sequencing to identify the endogenous targets of LARP4B, we found binding enrichment in 3′ untranslated region (UTR) and coding sequence (CDS) regions, most strongly the 3′ UTR (Fig. 3f and Extended Data Fig. 3b). *t*-Distributed stochastic neighbor embedding (*t*-SNE) clustering grouped LARP4B with other 3′ UTR-binding RBPs from the ENCODE (Encyclopedia of DNA Elements) project (Extended Data Fig. 3c), and motif analysis revealed poly(A) and poly(U) tracts (Fig. 3g), consistent with known AU-rich 3′ UTR-binding^41^. Transcripts with high LARP4B eCLIP enrichment (cutoff = 3) showed significantly higher translation efficiency (ribosome profiling; that is, Ribo-seq) than low-binding transcripts^40^, most strongly at the 3′ UTR (Extended Data Fig. 3d,e).

Consistent with most circRNAs being thought to be not or lowly translated^14^, LARP4B RIP–circRNA sequencing showed a lower fraction of BSJ reads in LARP4B immunoprecipitates compared with the inputs, indicating that fewer circRNAs associated with LARP4B than mRNAs (Extended Data Fig. 3g). The 404 high-confidence LARP4B-bound circRNAs (Extended Data Fig. 3h) showed enriched translation evidence from the TransCirc database^42^, particularly in ribosome and polysome profiling datasets (RP/PP; Extended Data Fig. 3i), supporting an association between LARP4B and endogenous circRNA translation, though the causality remains untested.

Given the preference of LARP4B for poly(A)/poly(U) tracts, we explored whether incorporating these sequences could enhance translation. We replaced the MS2 sequences in the exo-circFirefly reporter with poly(A) (49A, owing to limitation of oligonucleotide synthesis) or poly(U) (50U) sequences versus a random 50-base^9^ sequence. Interestingly, both 50U and 49A sequences increased the luciferase signal to 4–8-fold (Fig. 3i). Although other poly(A)/poly(U)-binding proteins may also contribute, it highlights the potential to simplify vector design using RNA aptamers to recruit endogenous RBPs, without a separate effector construct.

### RBM15 is a multifaceted regulator of endogenous circRNAs

**Fig. 4:**
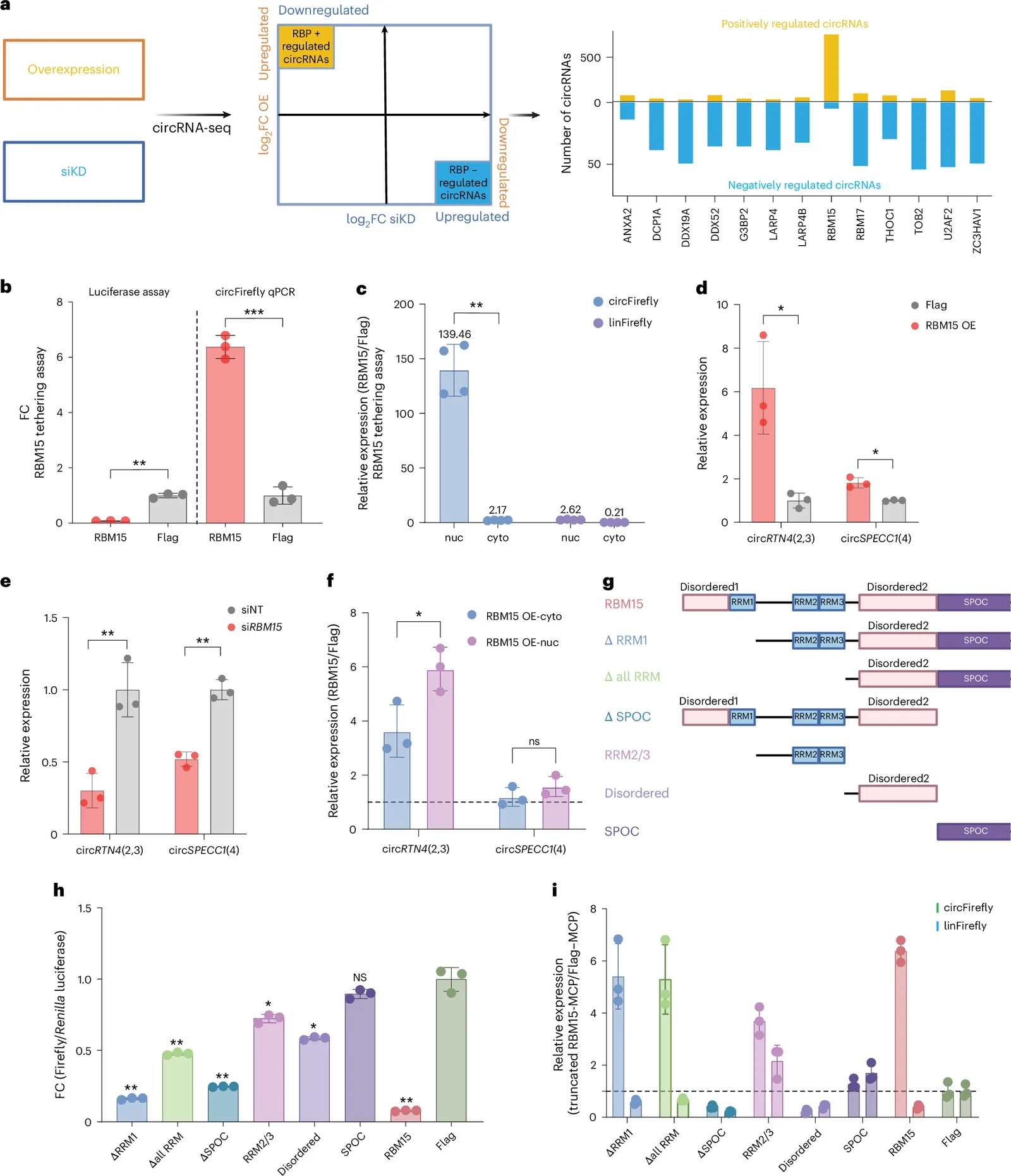
RBM15 is a multifaceted regulator of circRNAs. **a**, RBM15 positively regulates the expression of endogenous circRNAs. Overexpression (OE) and siRNA knockdown (siKD) of 13 RBP candidates followed by circRNA sequencing identifies RBM15 as a positive regulator of the expression of many endogenous circRNAs. **b**, RBM15 tethering on circFirefly reporter. Luciferase assay and qPCR results of RBM15 tethering on circFirefly reporter showing an increase of circFirefly biogenesis and a decrease in luciferase signal (mean ± s.d., *n* = 3). Dots represent individual biological replicates. Welch’s *t*-test, two-tailed. **P < 0.01; ***P < 0.001. **c**, Fractionation qPCR of circFirefly upon RBM15 tethering showing strong nuclear (nuc) retention of circFirefly RNA (mean ± s.d., *n* = 4). cyto, cytoplasmic. Dots represent individual biological replicates. Welch’s *t*-test, two-tailed. **P = 0.0014. **d**,**e**, Endogenous circRNA expression level upon RBM15 OE (**d**) and siRNA knockdown (si*RBM15*) (**e**) by qPCR (mean ± s.d., *n* = 3). Dots represent individual biological replicates. Welch’s *t*-test, two-tailed. *P < 0.05; **P < 0.01. **f**, Fractionation qPCR of endogenous circRNAs upon RBM15 OE, showing nuclear retention (mean ± s.d., *n* = 3). Dots represent individual biological replicates. Welch’s *t*-test, two-tailed. *P < 0.05. **g**, Schematic view of RBM15 domains and truncation constructs. **h**, Luciferase assay results showing the effect of truncated RBM15 tethering on circFirefly reporter (mean ± s.d., *n* = 3). Dots represent individual biological replicates.Welch’s *t*-test, two-tailed. *P < 0.05; **P < 0.01. **i**, Relative expression by qPCR showing the effect of truncated RBM15 tethering on circFirefly and linFirefly RNA level (mean ± s.d., *n* = 3). Dots represent individual biological replicates. Source data

**Extended Data Fig. 4:**
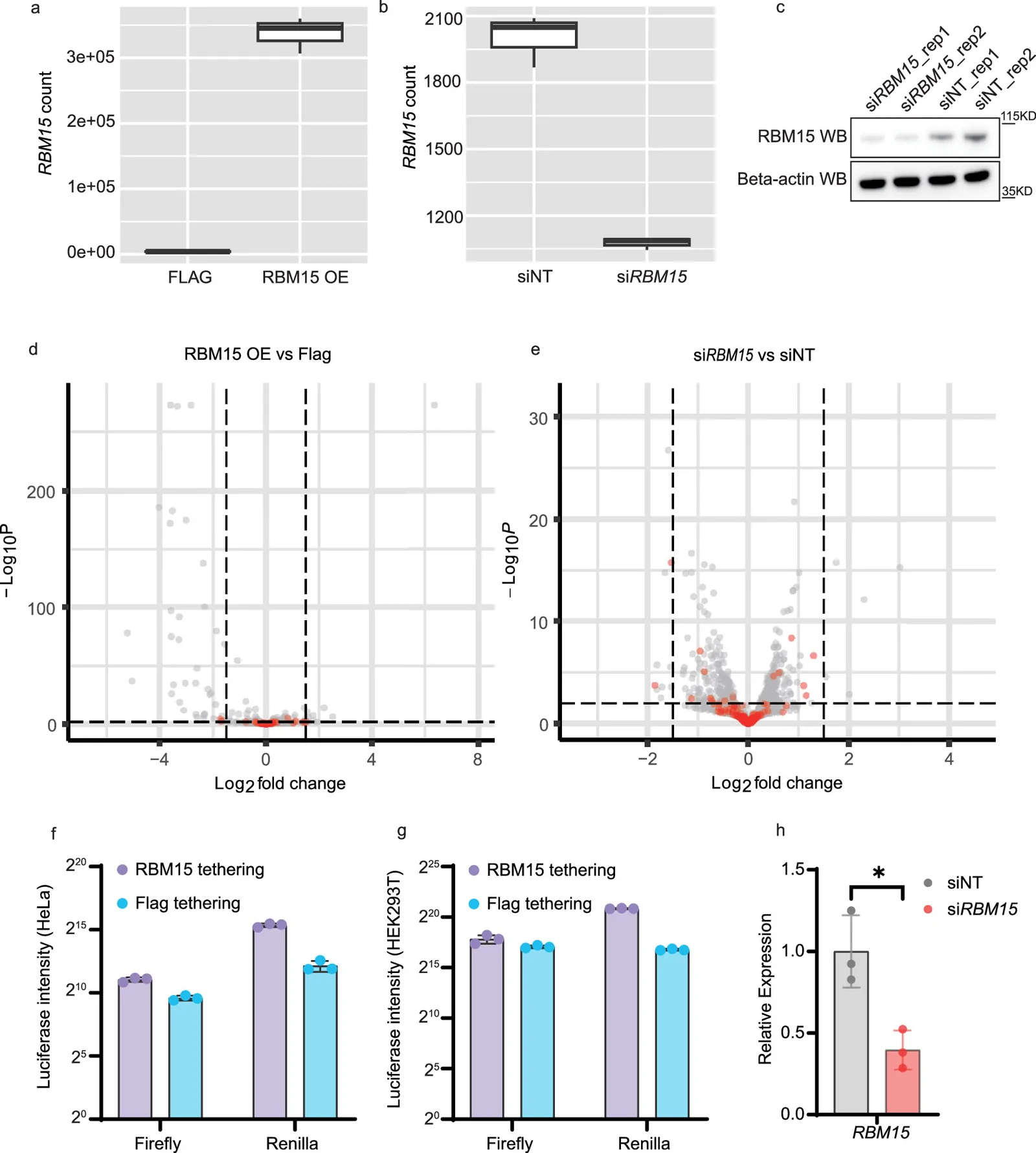
RBM15 is a multifaceted regulator of circRNA. **a**, **b**. Box plot of *RBM15* read counts showing successful overexpression and knockdown. **c**. Western blot showing successful knockdown of RBM15 at protein level by siRNA. Beta-actin is used as the reference gene. **d**, **e**. Volcano plot showing mRNA expression level changes upon RBM15 overexpression and knockdown. Red dots indicate the linear cognates of circRNAs that were positively regulated by RBM15, demonstrating that the circRNA changes are not due to transcription difference in the linear cognates. **f**, **g**. Bar plots showing raw luciferase signal of the circFirefly and circRenilla reporters upon RBM15 tethering assay (mean ± s.d., n = 3). Error bars represent standard deviations of biological replicates. **h**. Bar plot showing relative expression of *RBM15* upon siRNA knockdown of *RBM15* (mean ± s.d., n = 3). Error bars represent standard deviations of biological replicates. *: p < 0.05. Source data

To identify regulators of endogenous circRNAs, we performed gain- and loss-of-function studies (overexpression and siRNA knockdown) on 13 selected RBPs, classifying circRNAs upregulated by overexpression and downregulated by knockdown (or vice versa) as positively (or negatively) regulated (Fig. 4a). Translation-enhancing candidates (ANXA2, LARP4, LARP4B and G3BP2) did not broadly alter circRNA abundance, consistent with their primarily translational roles; nuclear export factors (THOC1 and DDX19A) and known/putative RNA degradation factors (TOB2, DCP1A and ZC3HAV1) each affected a limited subset of endogenous circRNAs (mostly downregulation for the latter, aligning with RNA decay function) (Fig. 4a and Supplementary Table 3). Surprisingly, RBM15 emerged as a strong positive regulator, influencing over 700 endogenous circRNAs (Fig. 4a and Supplementary Table 3). Ribosomal-RNA (rRNA)-depleted total RNA sequencing (RNA-seq) of RBM15 overexpression/knockdown samples (confirmed by *RBM15* read counts, Extended Data Fig. 4a,b, and at protein level, Extended Data Fig. 4c) showed minimal expression changes of their linear cognates (Extended Data Fig. 4d,e), confirming circRNA-specific regulation.

Revisiting the tethering assay, RBM15 tethering dramatically decreased relative luciferase signal while increasing circFirefly RNA (Fig. 4b). Given the known role of RBM15 in alternative splicing and exclusively nuclear localization, we hypothesized that it both promotes circRNA biogenesis and retains circRNAs in the nucleus, thereby reducing cytoplasmic signal. Indeed, RBM15 tethering increased nuclear circFirefly ~140-fold, but cytoplasmic circFirefly only 2.17-fold (Fig. 4c). Both Firefly (owing to tethering) and *Renilla* (owing to RBM15 overexpression) signals increased in raw data (Extended Data Fig. 4f,g). The stronger nuclear retention of circFirefly relative to circ*Renilla* explains the reduced cytoplasmic ratio and the net decrease in *Renilla*-normalized luciferase signal, together suggesting dual functions in circRNA biogenesis and nuclear retention.

On endogenous circRNAs, RBM15 overexpression significantly increased and knockdown significantly decreased circ*RTN4*(2,3) and circ*SPECC1*(4) levels (Fig. 4d,e; knockdown confirmed in Extended Data Fig. 4h). Fractionation revealed greater nuclear than cytoplasmic upregulation for both, suggesting that RBM15 also retains endogenous circRNAs in the nucleus, albeit less pronounced than for the tethered circFirefly reporter (Fig. 4f), possibly reflecting the RNA-binding affinity of RBM15.

In summary, RBM15 promotes circRNA biogenesis while retaining circRNAs in the nucleus, both for the reporter and endogenously.

### Different domains of RBM15 are responsible for its multifaceted functions

We next dissected the RBM15 functional domains: three RNA recognition motif (RRM) domains, one SPOC domain and two longer disordered domains, before RRM1 (Disordered1) and between the third RRM and the SPOC domain (Disordered2). We generated truncation constructs (ΔRRM1, Δall RRM, ΔSPOC, RRM2/3, disordered and SPOC; Fig. 4g), each fused to MCP and tested in the tethering assay.

Full-length RBM15 tethering decreased linear Firefly (linFirefly) RNA levels and increased circFirefly RNA levels (biogenesis), while reducing the relative luciferase signal (nuclear retention). Disordered1 and RRM1 domains were dispensable for both functions, as the ΔRRM1 construct behaved like full-length RBM15 (Fig. 4h,i). RRM2 and RRM3 domains were dispensable for biogenesis, as the Δall RRM construct still decreased linFirefly RNA and increased circFirefly RNA, though they may still contribute to specificity of endogenous binding sites. The partial recovery of luciferase activity suggested that RRM2 and RRM3 contribute to circRNA nuclear retention. Consistent with this, tethering RRM2/3 domains alone also reduced luciferase signal despite increasing circFirefly RNA, supporting their role in nuclear retention (Fig. 4h,i). Disordered2 and SPOC domains worked synergistically to promote biogenesis, as neither alone increased circFirefly RNA levels. The SPOC domain additionally regulated transcription, as Δ SPOC reduced and SPOC-alone tethering increased linFirefly RNA (Fig. 4h,i). These findings are consistent with its interaction with SETD1B to regulate transcription^43^. In conclusion, distinct RBM15 domains separately drive circRNA biogenesis and nuclear retention.

### RBM15 directly regulates circRNA biogenesis

**Extended Data Fig. 5:**
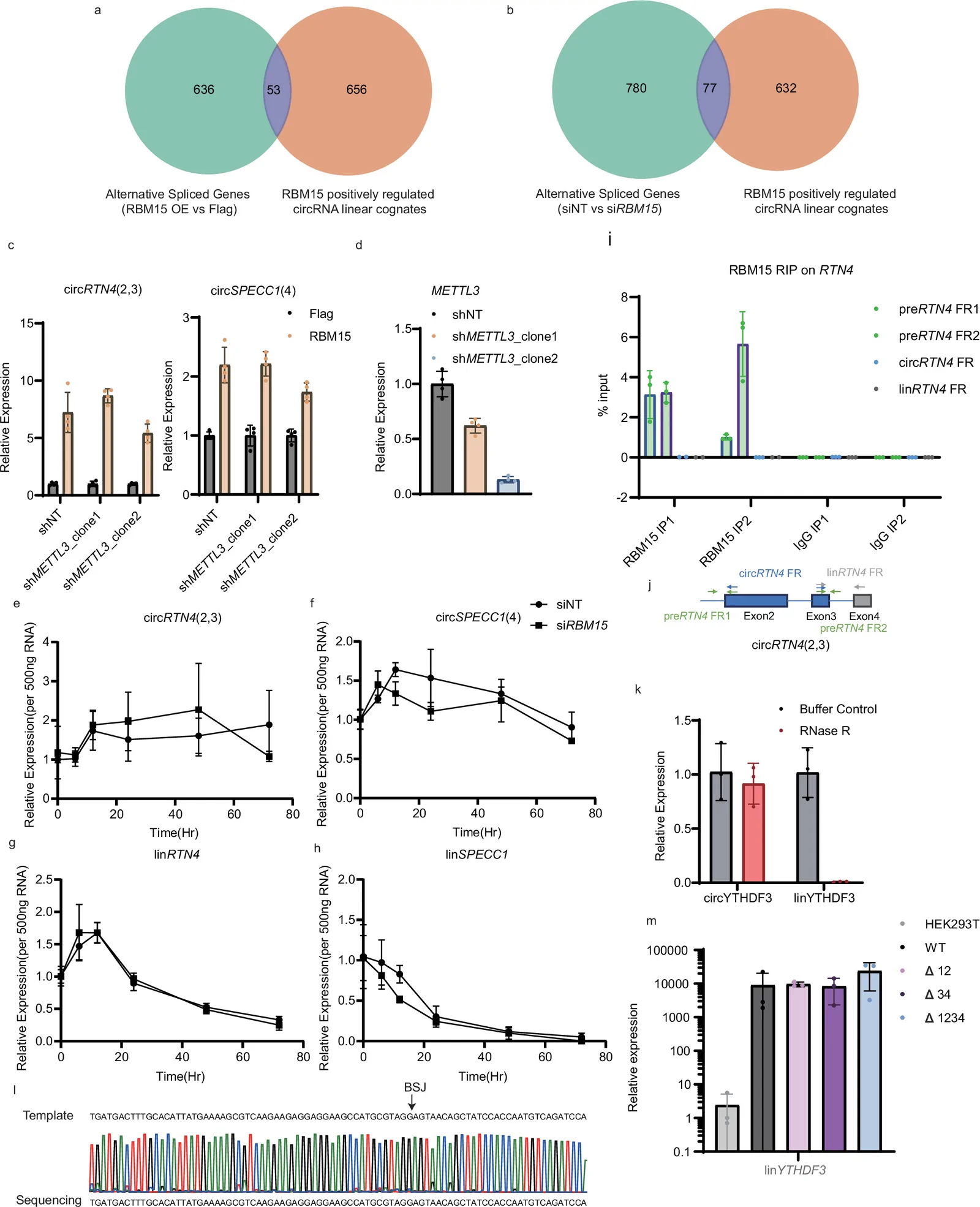
RBM15 directly regulates circRNA biogenesis. **a**, **b**. Venn Diagram showing minimal gene level overlap between RBM15 regulated circRNAs and RBM15 regulated exon skipping events. **c**. Bar plot showing relative expression of endogenous circRNAs upon RBM15 overexpression in *METTL3* knockdown stable cell lines (mean ± s.d., n = 4). Error bars represent standard deviations of biological replicates. **d**. Bar plot showing relative expression of *METTL3* in *METTL3* knockdown stable cell lines (mean ± s.d., n = 4). Error bars represent standard deviations of biological replicates. **e**-**h**. Stability measurement of circRNAs and their linear cognates upon the depletion of RBM15, showing no difference in stability (mean ± s.d., n = 3). Error bars represent standard deviations of biological replicates at each time point. Each RNA is normalized to time 0. **i**. RIP-qPCR showing enriched binding of RBM15 to the precursor mRNA where circ*RTN4*(2,3) originated from (mean ± s.d., n = 3). Error bars represent standard deviations of technical replicates. **j**. Schematics showing primer design for qPCR of precursor mRNA, mature mRNA and mature circRNA from *RTN4*. **k**. Bar plot showing the RNA level comparison of circ*YTHDF3*(3) and lin*YTHDF3* before and after RNase R treatment by qPCR (mean ± s.d., n = 3). Error bars represent standard deviations of technical replicates. **l**. Nanopore sequencing of circ*YTHDF3*(3) qPCR product showing correct backsplicing junction sequence. **m**. Bar plot showing similar level of lin*YTHDF3* expression of the *YTHDF3* minigene and mutants (mean ± s.d., n = 3). Error bars represent standard deviations of biological replicates. Source data

To determine how RBM15 regulates circRNA biogenesis, we examined its known functions in alternative splicing^44^ and m^6^A modification^45^. Splicing analysis revealed predominant exon-skipping events regulated by RBM15, with minimal overlap with the parental genes of circRNAs that RBM15 positively regulated (Extended Data Fig. 5a,b). Furthermore, in METTL3-depleted cells (Extended Data Fig. 5d), circRNA upregulation persisted at levels similar to those in control cells, together ruling out both functions as contributors (Extended Data Fig. 5c).

Both circ*RTN4*(2,3) and circ*SPECC1*(4) were highly stable over 72 h (Extended Data Fig. 5e,f) relative to their linear cognates (Extended Data Fig. 5g,h), consistent with circRNAs’ known greater stability^46^. The depletion of RBM15 did not alter degradation kinetics of both linear and circular RNA, ruling out RNA stabilization as its mechanism (Extended Data Fig. 5e–h).

**Fig. 5:**
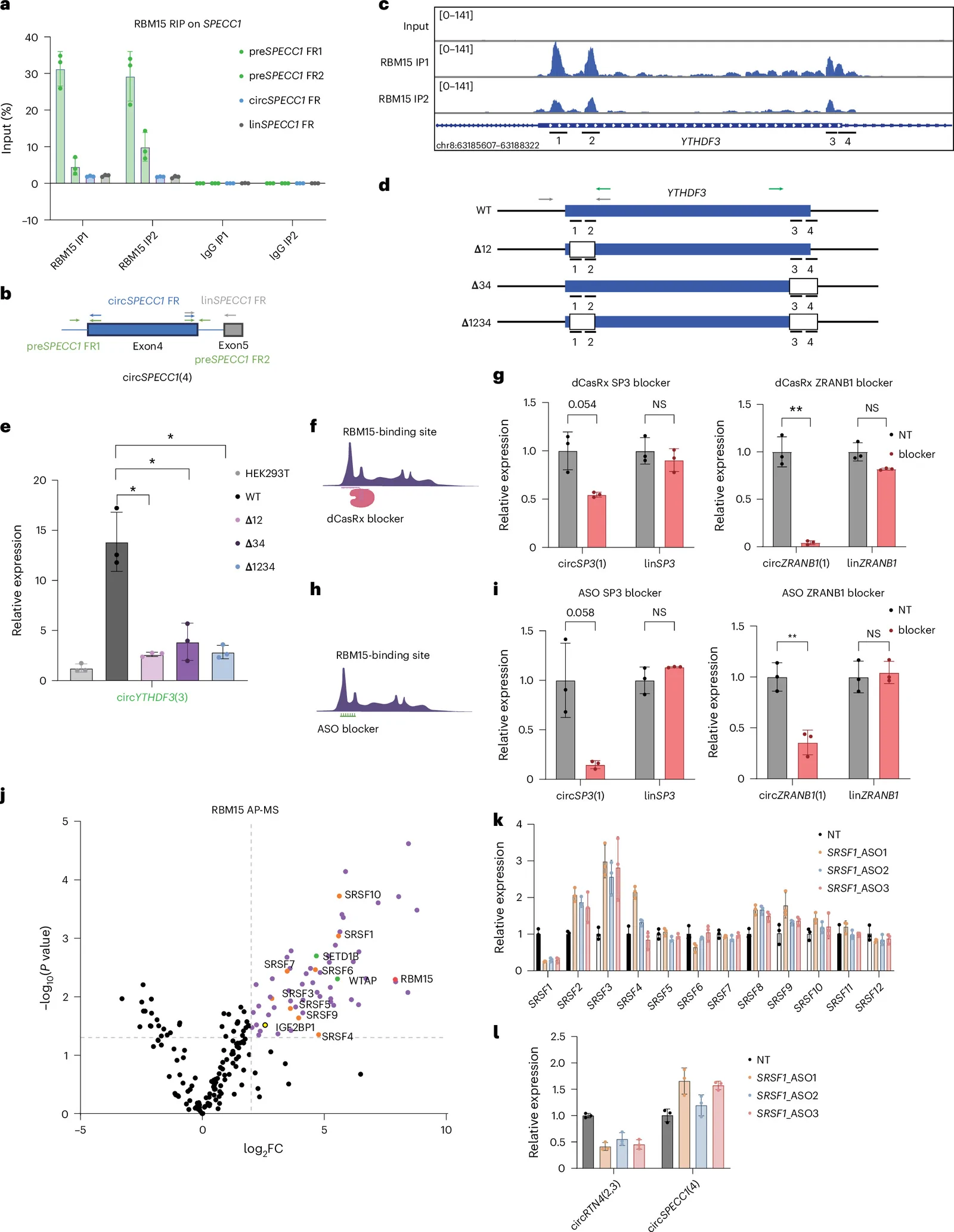
RBM15 binds pre-mRNA and regulates circRNA biogenesis. **a**, RIP–qPCR showing enriched binding of RBM15 to the precursor mRNA where circ*SPECC1*(4) originated from (mean ± s.d., *n* = 3). Dots represent technical replicates. Two biological replicates are shown as IP1 and IP2. FR, forward and reverse primer pair. **b**, Schematics showing primer design for qPCR of precursor mRNA, mature mRNA and mature circRNA from *SPECC1*. **c**, Integrative Genomics Viewer tracks showing RBM15-binding sites on *YTHDF3* exon 3. **d**, Schematic design of *YTHDF3* minigene and RBM15 binding site mutants. **e**, Bar plot showing qPCR detection of circ*YTHDF3*(3) expression level upon deletion of RBM15-binding sites (mean ± s.d. of biological replicates, *n* = 3). Welch’s *t*-test, two-tailed. *P < 0.05. WT, wild-type. **f**, Schematics of blocking RBM15-binding site with dCasRx. **g**, Bar plot showing successful inhibition of circ*SP3*(1) and circ*ZRANB1*(1) expression detected by qPCR (mean ± s.d. of biological replicates, *n* = 3). NT, non-targeting. Welch’s *t*-test, two-tailed. **P < 0.01; NS, *P* > 0.05. **h**, Schematics of blocking RBM15 binding site with ASO. **i**, Bar plot showing successful inhibition of circ*SP3*(1) and circ*ZRANB1*(1) expression detected by qPCR (mean ± s.d. of biological replicates, *n* = 3). Welch’s *t*-test, two-tailed. **P < 0.01; NS, *P* > 0.05. **j**, Volcano plot of RBM15 AP-MS result without RNase treatment. Purple, orange, green and yellow dots represent proteins passing both log_2_FC (>2) and *P* value (<0.05) cutoffs. Multiple *t*-test with *P* value correction. Green dots represent well-known RBM15 interacting partners, WTAP and SETD1B. Orange dots represent SRSF splicing factors. Yellow dot represents IGF2BP1. **k**, Bar plot showing successful knockdown of *SRSF1* by gapmer ASOs targeting *SRSF1* and expression changes for other SRSF genes by qPCR (mean ± s.d. of biological replicates, *n* = 3). **l**, Bar plot showing expression level changes of circ*RTN4*(2,3) and circ*SPECC1*(4) upon the depletion of *SRSF1* by qPCR (mean ± s.d. of biological replicates, *n* = 3). Source data

RBM15 RIP–qPCR showed higher enrichment on the pre-mRNA precursors of circ*SPECC1*(4) and circ*RTN4*(2,3) compared with their mature circRNAs and mRNA cognates (Fig. 5a,b and Extended Data Fig. 5i,j), demonstrating direct pre-mRNA occupancy. To test necessity, we generated a circ*YTHDF3*(3) minigene, chosen for its sparse RBM15 eCLIP sites (Fig. 5c), and mutants with binding sites deleted (Δ12, Δ34 or Δ1234; site 4 spans the exon–intron junction, so we retained the essential ‘GGgt’ splice-site bases) (Fig. 5d). The wild-type minigene increased circ*YTHDF3*(3) ~15-fold over basal expression (circRNA confirmed by RNase R–qPCR and Nanopore sequencing; Extended Data Fig. 5k,l), while all three deletion mutants markedly reduced circ*YTHDF3*(3) without affecting lin*YTHDF3* expression; the milder effect from deleting weaker sites (3,4) supports a dose-dependent requirement for RBM15 binding in circRNA biogenesis (Fig. 5e and Extended Data Fig. 5m).

### Blocking RBM15-binding site as a strategy for circRNA interference

Current circRNA knockdown methods (shRNA, siRNA and CasRx)^47–50^ all target BSJs, limiting sequence choice. Because RBM15 binding drives circRNA biogenesis, we reasoned that blocking its binding sites could serve as an alternative knockdown strategy. We designed guide RNAs targeting the most enriched RBM15 binding sites on *SP3* exon 1 and *ZRANB1* exon 1, which generate circ*SP3*(1) and circ*ZRANB1*(1), respectively, to tether dCasRx and sterically block RBM15 binding (Fig. 5f). This indeed significantly decreased circ*SP3*(1) and circ*ZRANB1*(1) expression with little effect on their linear cognates (Fig. 5g), confirming the causality between RBM15 binding and circRNA biogenesis. It offers a programmable loss-of-function strategy potentially applicable beyond the approximately 700 RBM15-regulated circRNAs already identified (see Discussion).

Steric-blocking ASOs targeting the same sites similarly decreased circ*SP3*(1) and circ*ZRANB1*(1) expression without affecting linear cognates (Fig. 5h,i), highlighting the therapeutic tractability of this strategy given the established clinical use of ASOs.

### RBM15 may cooperate with SRSF proteins to regulate circRNA biogenesis

**Extended Data Fig. 6:**
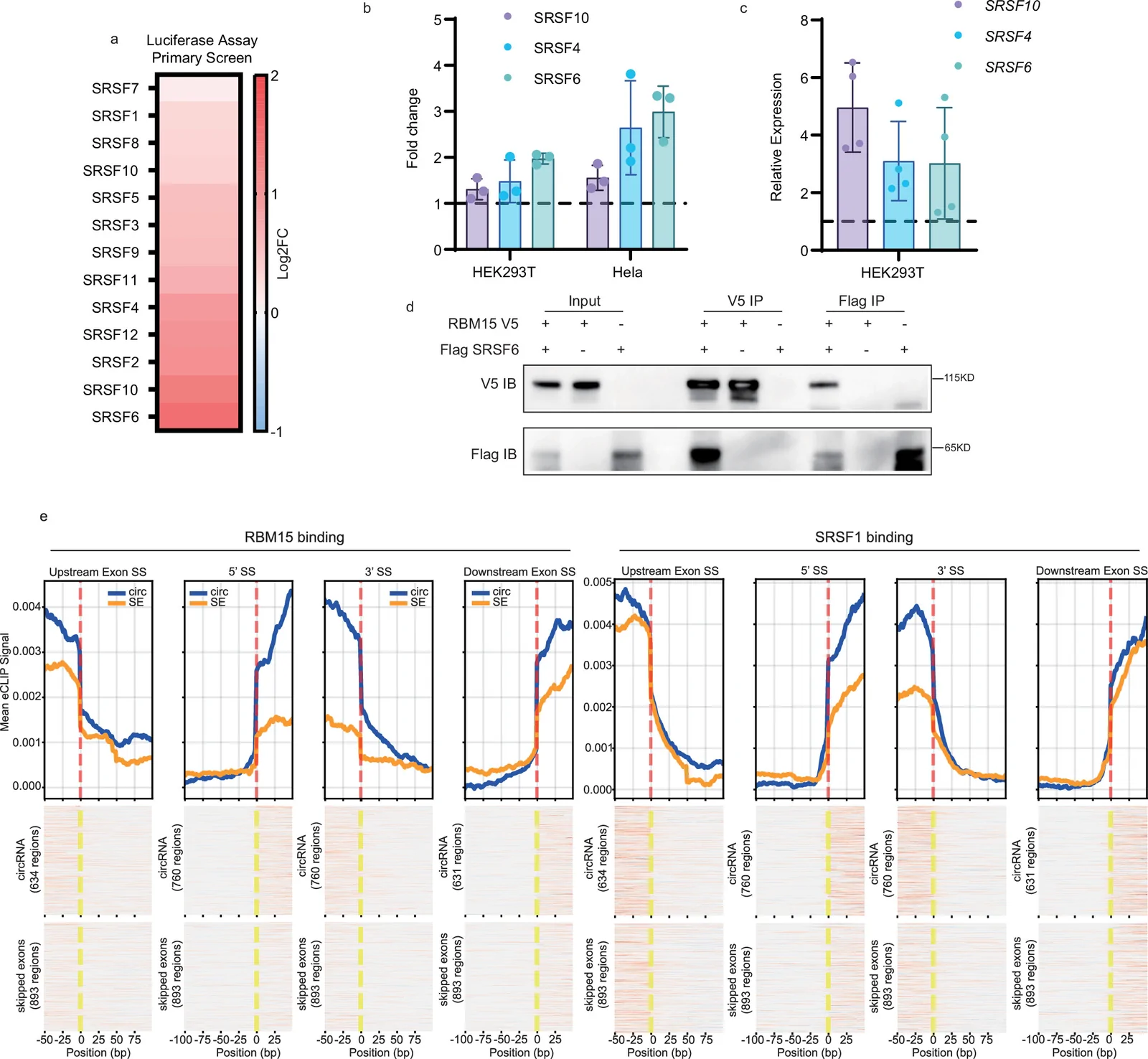
RBM15 may cooperate with SRSF proteins to regulate circRNA biogenesis. **a**. Heatmap of luciferase assay result from the primary tethering screen showing increased luciferase signals when SRSF splicing factors are tethered. **b**. Bar plot showing luciferase results of SRSF splicing factors from secondary screen in HEK293T and Hela cells (mean ± s.d., n = 3). Error bars represent standard deviations of biological replicates. **c**. Bar plot showing qPCR results of circFirefly upon SRSF splicing factors tethering from secondary screen in HEK293T cells (mean ± s.d., n = 4). Error bars represent standard deviations of biological replicates. **d**. Western blot showing successful co-immunoprecipitation between RBM15 and SRSF6 from the pull down of each protein. IB: immunoblot. **e**. Density plot and heatmap showing RBM15 and SRSF1 binding pattern on RBM15 regulated circRNAs or skipped exons. Density plot and heatmap were generated with normalized eCLIP binding data (IP subtracting input). Source data

Affinity pulldown followed by mass spectrometry (AP-MS) identified RBM15-interacting partners (Supplementary Table 4), including the known interactors WTAP (m^6^A writer complex)^51^ and SETD1B^43^ (Fig. 5j), and, notably, several SRSF splicing factors (Fig. 5j), confirmed by co-IP/western blot analysis for SRSF6 (Extended Data Fig. 6d). Tethering SRSF4, SRSF6 and SRSF10 increased both luciferase signal (secondary screen) and circFirefly RNA (Extended Data Fig. 6b,c), and other SRSF proteins also enhanced luciferase signal in the primary screen (Extended Data Fig. 6a), implicating that RBM15 may promote circRNA biogenesis through interactions with SRSF proteins.

Because SRSF proteins are densely auto- and cross-regulated^52^ and poorly targeted by antibodies, we examined SRSF1 with good eCLIP data from the ENCODE project^53^. The depletions of *SRSF1* by three ASO gapmers were all successful (Fig. 5k), but upregulated *SRSF2/3/8/9*, consistent with the dense cross-regulation (Fig. 5k). Interestingly, SRSF1 depletion decreased circ*RTN4*(2,3) but increased circ*SPECC1*(4) (Fig. 5l), possibly owing to compensatory SRSF upregulation, suggesting that different SRSF proteins may cooperate with RBM15 on different circRNA targets.

RBM15 and SRSF1 both bound with highest density near BSJs (Extended Data Fig. 6e). For RBM15-regulated circRNAs and skipped exons, both proteins bound heavily at the upstream/downstream exon boundaries, with similar binding at the circRNAs but lower binding at the skipped exons—a pattern that may determine whether RBM15 drives exon skipping versus backsplicing.

### RBM15 retains IGF2BP1 in the nucleus

**Fig. 6:**
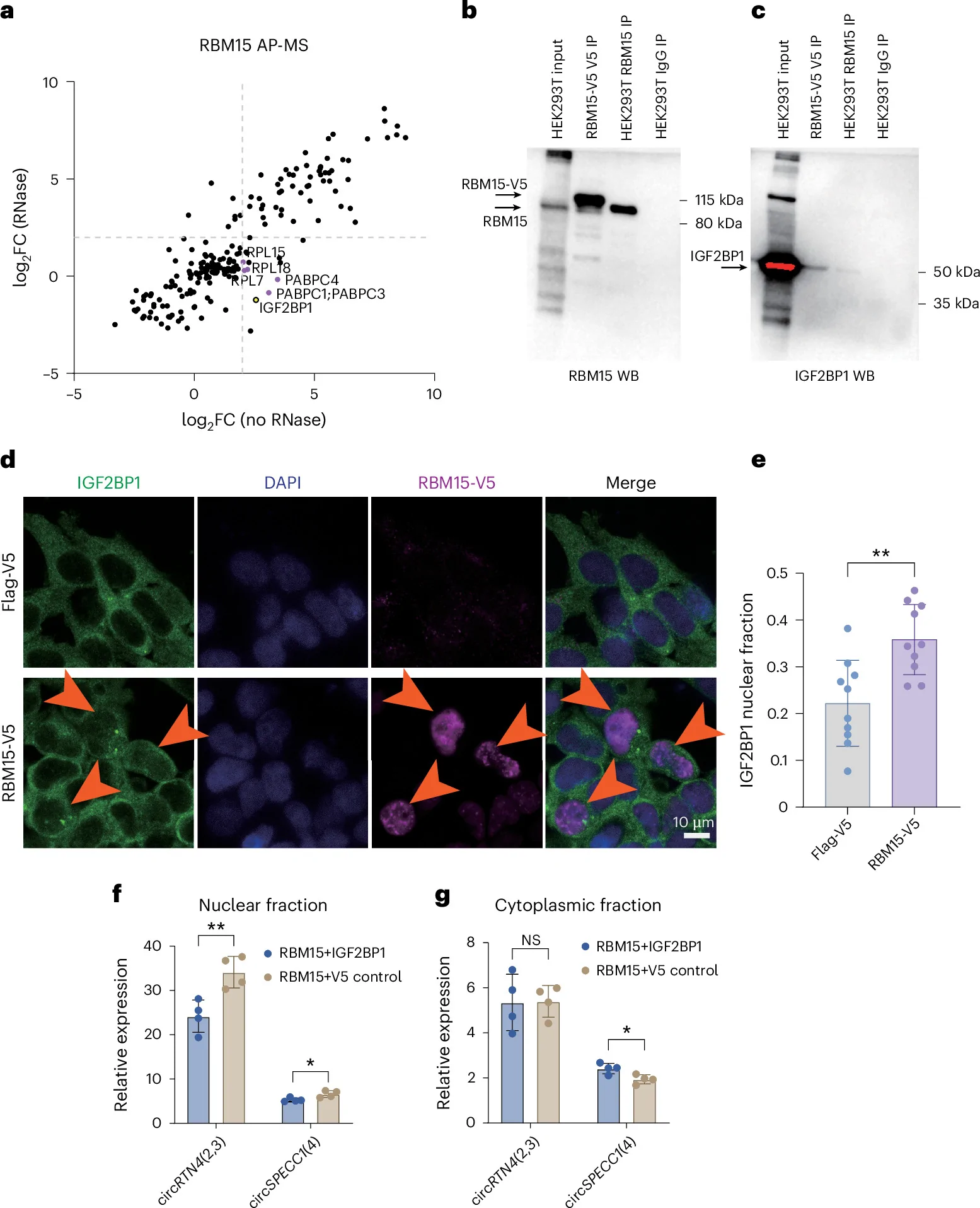
RBM15 retains IGF2BP1 in the nucleus. **a**, Scatter plot comparing RBM15 AP-MS data with and without RNase treatment. Dash lines represent the log_2_FC cutoff. RBM15 interacting partners that are only significant without RNase treatment are highlighted in purple. IGF2BP1 is highlighted in yellow. **b**, RBM15 western blot (WB) showing successful pulldown of RBM15 by V5 tag or anti-RBM15 antibody. The validation co-IP was not repeated but cross-validated with AP-MS. **c**, IGF2BP1 western blot showing the pulldown of IGF2BP1 from RBM15 co-IP experiments. The validation co-IP was not repeated but cross-validated with AP-MS. **d**, Immunofluorescence showing nuclear retention of IGF2BP1 protein upon RBM15 OE. The arrowheads highlight cells with higher RBM15 expression and more IGF2BP1 nuclear retention. Green: IGF2BP1; blue: DAPI; magenta: RBM15-V5. Scale bar, 10 μm. **e**, Quantification of immunofluorescence images (mean ± s.d. of 10 imaging fields from each group, *n* = 10). Welch’s *t*-test, two-tailed. **P = 0.002. **f**, Bar plot showing partial rescue of circRNA nuclear retention by co-expressing IGF2BP1, detected by qPCR (mean ± s.d. of biological replicates, *n* = 4). Welch’s *t*-test, two-tailed. **P = 0.008; *P = 0.033. **g**, Bar plot showing minimal change or slight increase of the cytoplasmic fraction by co-expressing IGF2BP1, detected by qPCR (mean ± s.d. of biological replicates, *n* = 4). Welch’s *t*-test, two-tailed. *P = 0.022; NS, *P* > 0.948. Source data

**Extended Data Fig. 7:**
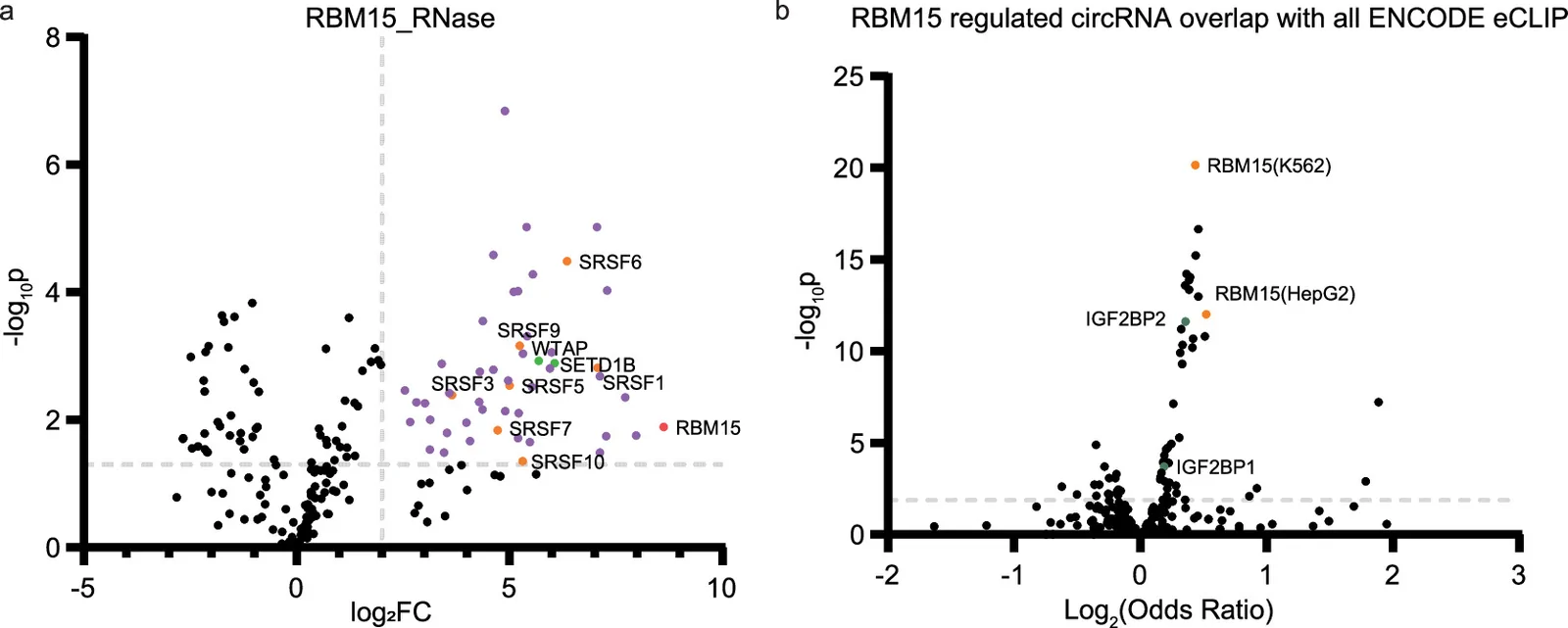
RBM15’s interaction with IGF2BP1 is RNA-dependent. **a**. Volcano plot of RBM15 AP-MS result with RNase treatment. Purple, orange and green dots represent proteins passing both log_2_FC (>2) and p value (<0.05) cutoffs. Multiple t-test with p value correction. Green dots represent well known RBM15 interacting partners, WTAP and SETD1B. Orange dots represent SRSF splicing factors. **b**. Volcano plot showing the overlap between RBM15 positively regulated circRNAs and all ENCODE eCLIP datasets. Multiple Chi-square test with p value correction. Each dot represents one eCLIP experiment. RBM15 is highlighted in orange and IGF2BP1 and IGF2BP2 proteins are highlighted in blue. Source data

To probe the potentially RNA-dependent nuclear retention function of RBM15 (Fig. 4c,f,h,i), we performed AP-MS with and without RNase A treatment. RNase A treatment markedly reduced RBM15–IGF2BP1 interaction, indicating RNA-mediated binding (Fig. 6a). We confirmed RBM15–IGF2BP1 interaction by co-IP both with V5-tagged and endogenous RBM15 (Fig. 6b,c). Notably, interactions with WTAP, SETD1B and SRSF proteins persisted following RNase A treatment (Extended Data Fig. 7a), consistent with the RRM-independent biogenesis function of RBM15.

Considering that IGF2BP1 functions as a circRNA nuclear export adapter^27^, we asked whether RBM15 retains IGF2BP1—and thereby circRNAs—in the nucleus. Indeed, immunofluorescence revealed significantly higher nuclear localization of IGF2BP1 upon RBM15 overexpression, scaling with RBM15 transgene expression level (Fig. 6d,e), supporting the sequestration of IGF2BP1. RBM15-regulated circRNAs were significantly enriched for both RBM15 and IGF2BP1 binding sites, potentially scaffolding this interaction (Extended Data Fig. 7b).

To illustrate dependence on IGF2BP1, we conducted a rescue assay by overexpressing IGF2BP1 in the context of RBM15 overexpression. At a titrated 1:9 (RBM15 to IGF2BP1) plasmid ratio, we observed a partial but significant rescue of the nuclear retention for both circ*RTN4*(2,3) and circ*SPECC1*(4) (Fig. 6f,g), suggesting that RBM15-mediated nuclear retention acts at least partly through IGF2BP1 sequestration. The large excess of IGF2BP1 needed to overcome retention underscores the importance of RBM15 dosage for circRNA homeostasis.

Together, these findings show that RBM15 retains IGF2BP1 in the nucleus, contributing to circRNA nuclear retention, likely through shared binding sites to scaffold their interaction.

### RBM15 binds directly to the exonic region near BSJs and is a conserved circRNA regulator

**Fig. 7:**
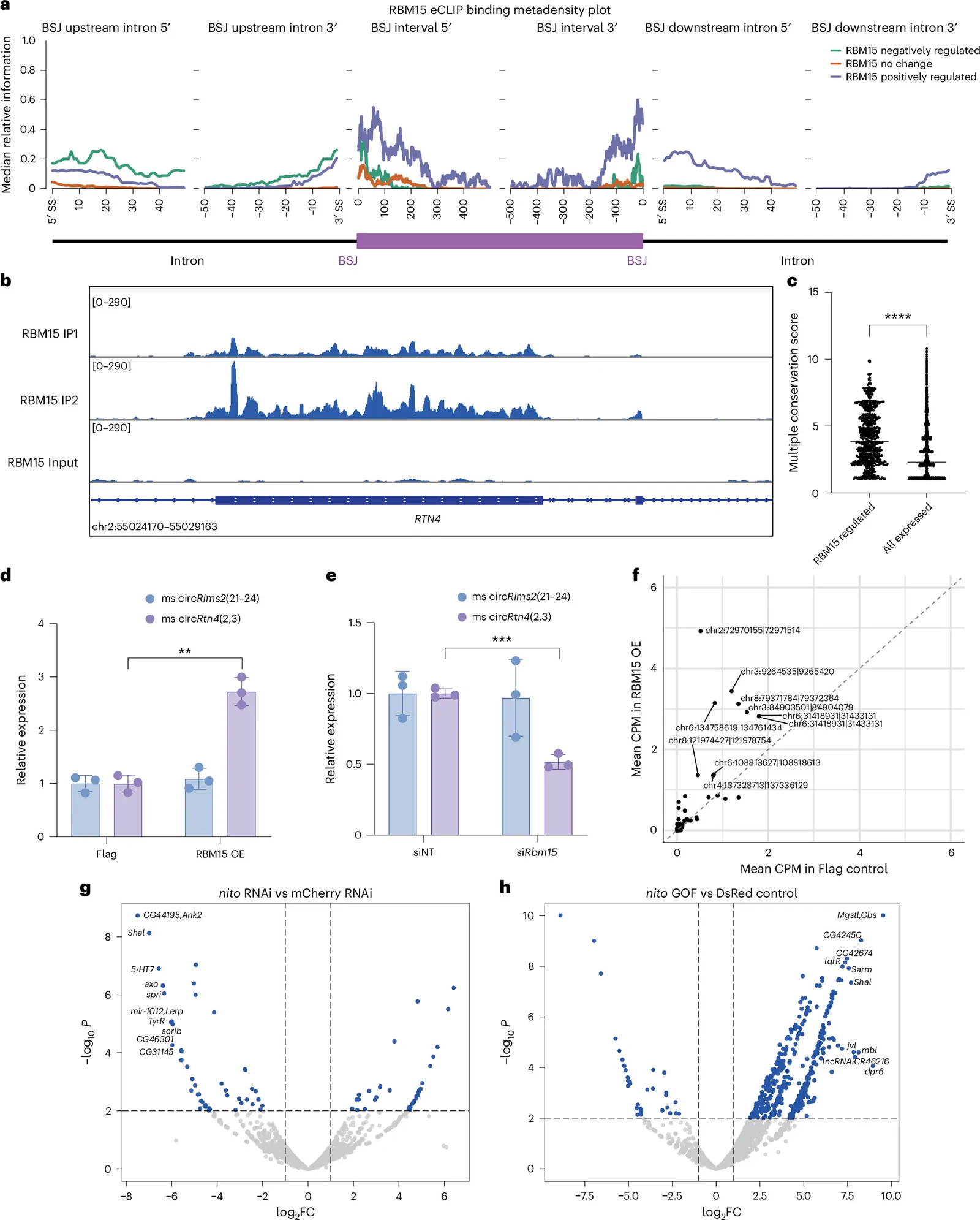
RBM15 is a conserved circRNA regulator. **a**, Metadensity plot of RBM15 eCLIP binding on circRNAs showing enriched binding of RBM15 near the BSJs of the circRNAs that it positively regulates. **b**, Integrative Genomics Viewer tracks showing RBM15 binding on *RTN4* exons 2 and 3. **c**, Comparison of the conservation between RBM15 positively regulated circRNAs and all circRNAs expressed in HEK293T cells. The line represents the median of the MCS from each group. Welch’s *t*-test, two-tailed. ****P < 0.0001. **d**,**e**, Relative expression of mouse (ms) circRNAs upon RBM15 OE (**d**) and siRNA knockdown (**e**) in N2A cells (mean ± s.d of biological replicates., *n* = 3). Welch’s *t*-test, two-tailed. ***P < 0.001; **P < 0.01. **f**, Scatter plot showing the mean CPM of three biological replicates in RBM15 OE samples versus Flag control samples. Each dot represents one circRNA. **g**, Volcano plot showing differential circRNA expression analysis between *nito* RNAi (*n* = 3) and mCherry RNAi (as control, *n* = 3) in flies. Significantly differentially expressed circRNAs (|log_2_FC| > 1 and *P* < 0.01) are highlighted blue. *P* values are from statistical analysis by ciri quant. Top 10 downregulated circRNAs by FC are labeled with the gene name where the circRNAs originate from. **h**, Volcano plot showing differential circRNA expression analysis between *nito* GOF (*n* = 4) and DsRed OE (as control, *n* = 3) in flies. Significantly differentially expressed circRNAs (|log_2_FC| > 1 and *P* < 0.01) are highlighted blue. *P* values are from statistical analysis by ciri quant. Top 10 upregulated circRNAs by FC are labeled with the gene name where the circRNAs originate from. Source data

**Extended Data Fig. 8:**
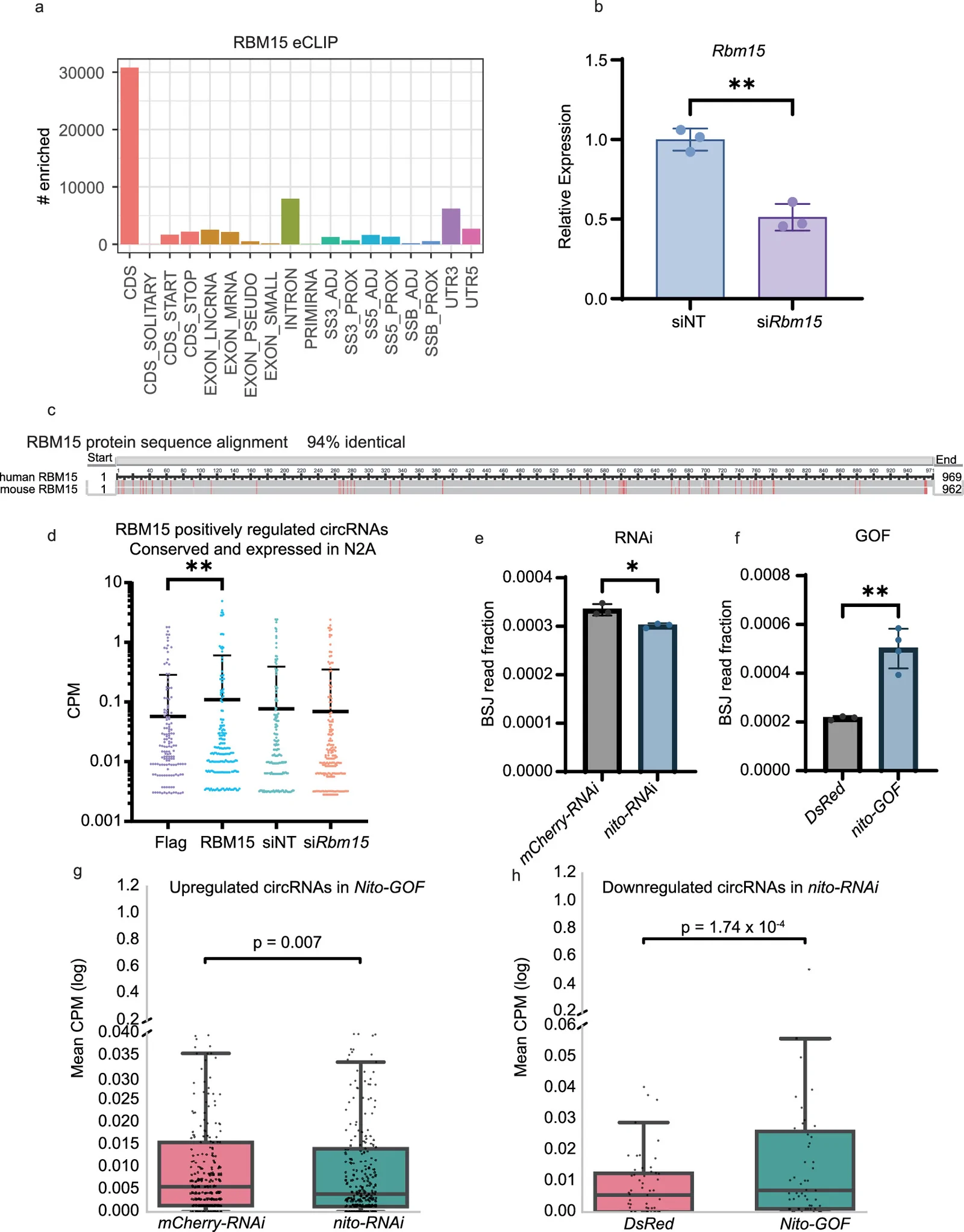
RBM15 is a conserved regulator of circRNA biogenesis. **a**. Bar plot showing region distribution of RBM15 eCLIP binding sites. **b**. Bar plot showing relative expression of *Rbm15* in N2A cells with *Rbm15* knockdown by siRNA (mean ± s.d., n = 3). Error bars represent standard deviations of biological replicates. Weich’s t-test, two-tailed. **: p < 0.01 **c**. Protein sequence alignment between human and mouse RBM15 proteins. Red color highlights the mismatches. **d**. Box plot of circRNA expression in N2A cells showing conserved circRNAs positively regulated by RBM15. Y axis is presented in log scale. The mid line shows the mean of each group. Error bars represent standard deviations of all conserved RBM15 positively regulated circRNAs. Weich’s t-test, two-tailed. **: p < 0.01 **e**. Bar plot showing BSJ read fraction from rRNA-depleted total RNA sequencing of fly RNAi samples, indicating total level decrease by the depletion of nito (mean ± s.d., n = 3). Error bars represent standard deviations of biological replicates. Weich’s t-test, two-tailed. *: p < 0.05 **f**. Bar plot showing BSJ read fraction from rRNA-depleted total RNA sequencing of fly GOF samples, indicating total level increase by the overexpression of nito (mean ± s.d., n = 3). Error bars represent standard deviations of biological replicates. Weich’s t-test, two-tailed. **: p < 0.01 **g**. Box plot showing CPM (log(Mean)) comparison between Control (mCherry) RNAi and Nito RNAi on significantly upregulated circRNAs in Nito GOF samples. Wilcoxon signed-rank test. **h**. Box plot showing CPM (log(Mean)) comparison between Control (dsRed) RNAi and Nito GOF on significantly downregulated circRNAs in Nito RNAi samples. Wilcoxon signed-rank test. Source data

Re-examination of RBM15 eCLIP data revealed predominant CDS binding (Extended Data Fig. 8a). A metadensity plot centered on BSJs showed increased RBM15 binding at exons flanking BSJs of positively regulated circRNAs versus unaffected ones (Fig. 7a), exemplified by circ*RTN4*(2,3) (Figs. 7b and 4d,e). Because exonic sequences are evolutionarily more conserved than introns, we hypothesized that the regulatory role of RBM15 might also be conserved across species. In contrast to ZC3H14^21^, the only other known exonic circRNA regulator, RBM15 binds more extensively across exonic regions, potentially aiding recognition of conserved sequence elements.

Human and mouse RBM15 proteins shared 94% sequence identity (Extended Data Fig. 8c), and RBM15 positively regulated circRNAs showed higher multiple conservation scores (MCS) reflecting the number of species, tissues and individuals sharing a given circRNA^54^. In mouse N2A cells, the conserved circ*Rtn4*(2,3)^55^ increased upon RBM15 overexpression and decreased upon *Rbm15* knockdown, while a non-RBM15-regulated circRNA (circ*Rims2*(21–24)) was unaffected (Fig. 7d,e; knockdown confirmed in Extended Data Fig. 8b). Lifting over RBM15-regulated circRNAs in HEK293T cells to the mouse genome annotated 730 of 756 circRNAs using circPrimer2.0 (ref. ^56^; Supplementary Table 5), of which 357 circRNAs were expressed in N2A cells. Average counts per million reads (CPM) of these circRNAs significantly increased upon mouse RBM15 overexpression (Fig. 7f and Extended Data Fig. 8d). These findings define RBM15 as a conserved regulator of circRNA biogenesis, likely via direct binding to conserved exonic BSJ-flanking sequences.

**Extended Data Fig. 9:**
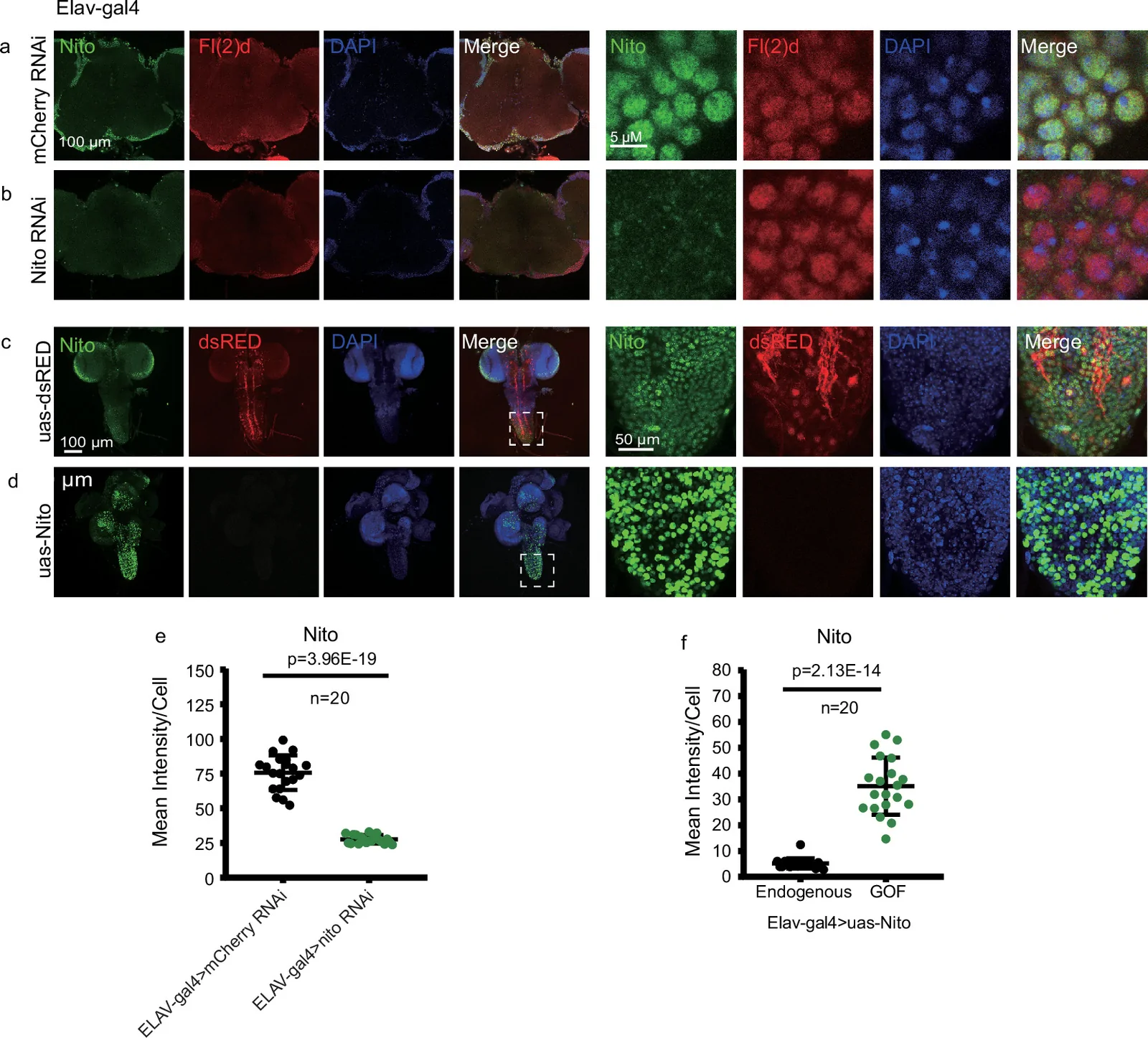
Validation of Nito knockdown and overexpression. **a-b**. Immunofluorescence showing successful depletion of Nito protein in fly heads under the respective RNAi conditions. Blue: DAPI; Green: Nito; Red: Fl(2)d. **c-d**. Immunofluorescence showing successful overexpression of Nito protein in 3^rd^ instar larvae under the respective overexpression conditions. Blue: DAPI; Green: Nito; Red: dsRed. **e**. Quantification of mean intensity of Nito protein under the respective RNAi conditions (mean ± s.d., n = 20). Error bars represent standard deviations of biological replicates. Weich’s t-test, two-tailed. **f**. Quantification of mean intensity of Nito in endogenous and overexpressed cells in Nito overexpression condition (mean ± s.d., n = 20). Error bars represent standard deviations of biological replicates. Weich’s t-test, two-tailed. Source data

To test conservation in an invertebrate, we used the Gal4-UAS (upstream activating sequence) system in *Drosophila* to both deplete Nito using transgenic RNA interference (RNAi) and overexpress myc-tagged Nito (gain-of-function, GOF). We confirmed successful knockdown and overexpression of Nito protein using immunostaining and image quantification (Extended Data Fig. 9a–f). Using *elav-Gal4* to manipulate Nito levels primarily in neurons, *nito* RNAi adults died after 1–2 days, while *nito* GOF animals died at second to third instar larval stages, indicating that precise *Drosophila nito* dosage is essential for viability.

We then conducted rRNA-depleted total RNA-seq analysis of both neuronal RNAi and GOF manipulations of Nito, compared with their respective control knockdown (mCherry RNAi) and overexpression (DsRed) genetic controls. As levels of circRNAs are well-known to increase with age^57,58^, we elected to analyze the latest possible stages for these manipulations (that is, 1–2-day adults for knockdown, and third instar larvae for GOF). The fraction of BSJ reads significantly decreased in RNAi samples and dramatically increased in GOF samples, consistent with biogenesis-promoting function (Extended Data Fig. 8e,f). Differential expression analysis (accounting for linear cognate expression changes) showed more downregulated (62) versus upregulated circRNAs (36) with *nito-RNAi*, and a much larger, opposite shift with *nito* GOF (517 up versus 37 down) (Fig. 7g,h).

The weaker RNAi phenotype may reflect circRNA stability requiring more time to manifest than the short RNAi survival window allowed, incomplete (rather than null) knockdown or lower sensitivity of total RNA-seq versus RNase R-enriched circRNA sequencing. Nonetheless, circRNAs upregulated in *nito* GOF were significantly downregulated in *nito-*RNAi and vice versa (Extended Data Fig. 8g,h). This cross-experiment concordance suggests that the regulatory role of RBM15 in circRNA biogenesis is also conserved in flies.

Collectively, RBM15 is a conserved regulator of circRNA biogenesis across humans, mice and flies. The conserved sequences that RBM15 regulates between human and mouse offer unexpected insights into how circRNAs could be regulated across species.

## Discussion

We developed a tethering-based circRNA luciferase reporter system to screen 730 RBPs (846 isoforms) for effects on circRNA expression, identifying 267 isoforms that increased and 29 that decreased signal; a secondary screen in two cell lines confirmed these hits. We focused on two classes: RBPs that enhance circRNA translation, relevant to circRNA vaccines and therapeutics, and RBPs that regulate endogenous circRNAs, which inform circRNA biogenesis mechanisms.

Two RBPs, LARP4B and ANXA2, enhanced circRNA translation in our exogenous reporter: LARP4B broadly across IRES elements and cell lines, ANXA2 in a cell-type-dependent manner. A focused follow-up screen extended this to six additional RBPs, most active across multiple cell types. LARP4B was also associated with translation of its endogenous targets, though causality remains untested, a reflection that our screen can identify both engineering effectors and candidate endogenous regulators. When we compared plasmid-derived circRNAs with in vitro-synthesized circRNAs—the therapeutically relevant format, unlike the plasmid-based reporters used in prior studies of translation-enhancing elements such as m^6^A modification^39^ and structured elements^59^—we found both shared and distinct regulatory features. This is an important consideration for future translation-enhancement screens. Together, these results establish tethering as a versatile platform for identifying translation-enhancing RBPs and point to two therapeutic strategies: co-delivering RBP-expressing constructs with circRNA therapeutics, or engineering RNA aptamers into circRNAs to recruit endogenous RBPs.

Among endogenous circRNA regulators, we discovered an unexpected role of RBM15 separate from its known functions, serving as a multifaceted regulator that both promotes circRNA biogenesis and retains circRNAs in the nucleus. Using AP-MS, we identified interactions between RBM15 and SRSF splicing factors, which likely promote circRNA biogenesis, and its RNA-dependent interaction with IGF2BP1, a circRNA adapter protein that RBM15 retains in the nucleus to mediate circRNA retention. Although SRSF proteins were previously shown to inhibit circRNA biogenesis in fly cells^19^, recently both this study and another CRISPR screen identified some of them as positive regulators of circRNA biogenesis in mammalian systems, suggesting differences of circRNA regulation across species. We further revealed direct binding of RBM15 specifically near the BSJs of the circRNAs it regulates. Although RBM15 may form a dimer to bridge the BSJs, the dimerization may not be necessary at least for the circRNA reporter where RBM15 is tethered to the middle of an exon but still greatly promotes backsplicing. Further findings demonstrate RBM15 as a conserved exonic circRNA regulator in humans, mice and flies. This is in contrast to previously proposed conserved regulatory elements such as intronic repeats, which are only weakly conserved at the sequence level^55^. The functionality of nuclear retention also merits further study, considering the recent demonstration of the nucleocytoplasmic export of circRNAs affecting neuronal functions^60^.

RBM15 also suggests a practical strategy for modulating endogenous circRNA levels: blocking its binding sites with guide RNAs or ASOs could expand the targetable landscape for circRNA-directed intervention. The ~700 RBM15-regulated circRNAs identified here likely underestimate the true total, since our stringent overexpression/knockdown concordance criteria may miss lowly expressed circRNAs and our analysis was limited to HEK293T cells rather than tissue-specific contexts. More broadly, the tethering screen measures regulatory potential rather than endogenous mechanism: of the 13 RBPs tested for gain- and loss-of-function effects, only RBM15 substantially affected endogenous circRNAs, while the others—including the positive control YTHDF2^6^—influenced only a small subset. Nevertheless, from an engineering perspective the screen’s effectors are immediately usable, as we show for circRNA translation, and we expect it will similarly yield tools for regulating endogenous circRNA expression.

## Methods

### Generation of plasmid constructs

The circFirefly and circ*Renilla* reporter sequences were designed by splitting Firefly and *Renilla* luciferase genes at an AG | GA sequence (to mimic original circ*ZKSCAN1* BSJ) and flanking encephalomyocarditis virus IRES sequence. Additional multiple cloning sites were introduced before IRES for cloning 6xMS2 sequence into the circFirefly reporter. Both reporters without MS2 sequences were ordered from Twist Bioscience and cloned into the pTwist CMV vector.

The RBP-MCP tethering library was generated as previously described^25^. Detailed information of the screen results with unique identifiers (Well ID and NCBI accession) for isoforms can be found in Supplementary Tables 1 and 2. The RBM15 fragments and the negative zinc finger protein controls were cloned into the same vector by gateway cloning. The primers used to amplify RBM15 fragments were listed in Supplementary Table 6.

The exo-circFirefly and exo-circ*Renilla* reporters were generated by amplifying from circFirefly and circ*Renilla* reporters with overlap PCR and subcloned into circRNA-synIRES-R25-mNeonGreen plasmid^9^ replacing synIRES and mNeonGreen by Gibson assembly. circRNA-synIRES-R25-mNeonGreen was a gift from H. Chang (Addgene plasmid 188115; http://n2t.net/addgene:188115; RRID: Addgene_188115).

The overexpression plasmids were generated by cloning RBP open reading frames (ORFs) into pEF5/FRT/V5-DEST vector (Thermo Fisher, V602020) or pLVpuro-CMV-N-3xFlag^61^ (Addgene plasmid 123223, for cloning SRSF6 and co-IP with RBM15) using gateway cloning. pLVpuro-CMV-N-3xFlag was a gift from R. Ketteler (Addgene plasmid 123223; http://n2t.net/addgene:123223; RRID:Addgene_123223). For knockdown, shRNA plasmids were derived from the Mission lentiviral shRNA library (Sigma-Aldrich).

All plasmids were verified by Sanger (Azenta) or Nanopore sequencing (Plasmidsaurus). The reporter plasmid maps are available in Supplementary Table 7.

### Cell lines

HEK293T, HeLa and Neuro-2a cells were originally purchased from the American Type Culture Collection and were not further authenticated. HeLa cells were a gift from the Jamieson lab. Cells were routinely tested for mycoplasma contamination with a MycoAlert mycoplasma test kit (Lonza) and were found negative for mycoplasma.

### Luciferase reporter assay

HeLa or HEK293T cells were seeded in 96-well plates precoated with poly-d-lysine (Sigma-Aldrich) at 20 K cells per well. After 24 h, RBP-MCP, circFirefly and circ*Renilla* plasmids were co-transfected in a ratio of 6:1:1 (100 ng in total) with TransIT-LT1 (Mirus Bio) following manufacturers’ instructions. Cells were lysed and luciferase activity was measured with Dual-Luciferase Reporter Assay System (Promega) in a Spark Multimode Microplate Reader (Tecan), following the manufacturer’s instruction, 72 h post transfection. RBM15 fragment tethering was carried out in HEK293T cells following the same procedures.

For exogenous luciferase reporters, HeLa or HEK293T cells were seeded in 96-well plates precoated with poly-d-lysine at 20,000 cells per well. After 24 h, RBP-MCP mRNA, exo-circFirefly and exo-circ*Renilla* circRNAs were co-transfected in a ratio of 6:1:1 (100 ng in total) with jetMESSENGER mRNA transfection reagent (Polyplus) following the manufacturer’s instructions. Cells were lysed and luciferase activity was measured with Dual-Luciferase Reporter Assay System (Promega) in a Spark Multimode Microplate Reader (Tecan), following the manufacturer’s instruction, 48 h post transfection.

### Statistical analysis

Luciferase data were first normalized to the *Renilla* internal control by calculating the ratio of Firefly signal over *Renilla* signal. The ratios were further normalized to the average ratio of Flag control to calculate the fold change. Two-tailed Student’s *t*-test was performed to calculate *P* values and multiple test correction was implemented to control FDR.

### GO analysis

GO analysis of the candidates from the screen was performed using DAVID online tools^62,63^. Background genes were set as the full list of the RBP-MCP library. Top GO terms in the Biological Processes were shown in the enriched GO term dot plot.

### Protein network analysis

Protein network of the candidates from the screen was generated from the STRING database^64^. Only experimental and database evidence was used to build the network.

### mRNA and circRNA synthesis

The RBP-MCP DNA templates were linearized by PCR amplification and gel extraction. RBP-MCP mRNAs were produced with HiScribe T7 ARCA mRNA Kit (with tailing, NEB) with the addition of 5mCTP and Pseudo-UTP, following the manufacturer’s instructions. The concentration was measured by nanodrop and size was measured by RNA Tapestation (Agilent).

For exo-circFirefly and exo-circ*Renilla* circRNA production, the protocol was described previously^27^ with slight modifications. In brief, DNA templates were linearized by PCR amplification and gel extraction. circRNA precursors were synthesized from 100 to 200 ng of linearized DNA template in a 20 μl reaction using the HiScribe T7 High Yield RNA Synthesis Kit (NEB), following the manufacturer’s instructions. After transcription and DNase I digestion, 77 μl RNase-free water and 2 μl additional GTP (10 mM) were added to the reaction (total volume 100 μl), and the reaction was heated to 55 °C for 30 min. The RNA was purified with RNA Clean & Concentrator-25 (Zymo Research). To remove linear RNA, 20 μg RNA was resuspended into final volume of 86 μl RNase-free water and heated at 65 °C for 3 min and immediately placed on ice for 3 min. Linear RNA was removed by digestion with 20 U RNase R (Lucigen) for 15 min at 37 °C, with addition of further 10 U RNase R and 15 min incubation. RNA was then purified with RNA Clean & Concentrator-5. The concentration was measured by nanodrop and purity was assessed by RNA Tapestation.

### RNA fractionation

Fractionation of cytoplasmic and nuclear RNA was performed as previously described^65^ with slight modifications. In brief, 1 million cells were washed in 1× PBS and subsequently permeabilized in 50 μl of cold lysis buffer (10 mM Tris-HCl, pH 7.4, 10 mM NaCl, 3 mM MgCl_2_ and 0.1% IGEPAL CA-630) by gentle pipetting five times. The nuclei were centrifuged at 500*g* for 5 min at 4 °C. The supernatant was harvested as cytoplasmic fraction and mixed with 150 μl TRIzol LS and stored at −80 °C until RNA extraction. The nuclei were washed in 200 μl lysis buffer once, lysed in 200 μl TRIzol Reagent as the nuclear fractions and stored at −80 °C until RNA extraction. The performance of fractionation was tested by reverse transcription quantitative PCR (RT–qPCR) to measure the relative levels of *MALAT1* (nuclear) and *ACTB* (predominantly cytoplasmic) mature mRNA.

### RNA extraction, reverse transcription and qPCR

RNA samples were harvested in TRIzol Reagent or TRIzol LS Reagent and extracted with Direct-Zol RNA miniprep kit (Zymo Research), following the manufacturer’s instructions. RNA (500 ng) was reverse transcribed with iScript cDNA Synthesis Kit (Bio-Rad) with the modification of scaling down to 10 μl reaction volume following the manufacturer’s instructions. cDNA was diluted 10 times and 1 μl of the diluted cDNA was loaded as template for qPCR reactions with Power SYBR Green PCR Master Mix (Thermo Fisher) following manufacturer’s instructions. qPCR primers for linear mRNAs were designed by NCBI Primer Blast. qPCR primers for circRNAs were either designed by circPrimer2.0 or acquired from literature. All qPCR primers (together with circRNA chromosome coordinates) used are listed in Supplementary Table 6. For human cells, *HPRT* (total and cytoplasmic fraction) and *MALAT1* (nuclear fraction) were used as reference genes for normalization. For mouse cells, 18S rRNA was used as the reference gene for normalization.

### Statistical analysis

Relative expression was calculated with the ΔΔ*C*_t_ method. Statistical analysis of the qPCR results was performed on at least three biological replicates. One-tailed Student’s *t*-test was carried out to calculate *P* values.

### Immunofluorescence

Cells were fixed with cold 4% paraformaldehyde for 15 min, permeabilized with 0.2% Triton X-100 for 10 min and blocked with 7% fetal bovine serum (FBS) in PBS for 30 min at room temperature. After blocking, cells were incubated with anti-V5 antibodies (CST), or IGF2BP1 antibodies (CST) for 1 h at room temperature, followed by washing three times with PBS and incubation with Alexa 488 or Alexa 555 conjugated secondary antibodies (Invitrogen) for another 1 h. The cells were counterstained with 4′,6-diamidino-2-phenylindole (DAPI) for 5 min, washed three times with PBS and subjected to imaging. Images were acquired with either a Keyence BZ-X800 microscope, or a Zeiss LSM780 confocal microscope (for IGF2BP1 localization quantification).

### Imaging data analysis

Confocal images were processed and analyzed with Fiji (NIH)^66^. The ‘Threshold’ function was used to determine the average intensity of antibody or nanobody staining for the corresponding fluorescence channel; the threshold value was set to capture all cellular intensity of the antibody or nanobody. To determine the fraction of nuclear signal in each image, we created masks of the whole cell using the antibody or nanobody channel and masks of the nucleus using the DAPI channel. The DAPI mask was subtracted from the whole-cell mask to create a cytoplasm-only mask of each image. The masks were subtracted from the original image to remove extraneous intensity values, and the ‘Threshold’ function was used to determine the intensity of the remaining pixels. The total intensity (that is, area × intensity) of the nuclear antibody signal was divided by the total intensity of the whole cell to calculate the nuclear fraction. At least 400 cells were analyzed per condition. Student’s *t*-test was performed on the average nuclear fraction in 10 imaging fields of both groups.

### Western blot

Protein samples were denatured and reduced with NuPAGE LDS Sample Buffer (Invitrogen) and dithiothreitol (DTT) (Sigma-Aldrich). Electrophoresis was performed with NuPAGE Bis-Tris Mini Protein Gels (Invitrogen). Membrane transfer was performed in a wet transfer system at 30 V overnight. The membranes were blocked by 5% milk for 30 min, then incubated with primary antibody for 1 h, washed three times with Tris-buffered saline (TBS) + 0.05% Tween-20 (TBST), incubated with secondary antibody for 1 h and washed three times with TBST. The signal was then developed with SuperSignal West Femto Maximum Sensitivity Substrate and imaged in a gel imaging system (Azure). Antibodies used in this study are listed in Supplementary Table 6.

### siRNA knockdown

ON-TARGETplus siRNAs were purchased from Horizon Discovery as pools of four siRNAs for each gene knockdown. siRNAs were transfected with DF1 transfection reagent (Horizon Discovery) following the manufacturer’s instructions using reverse transfection protocol. Cells were obtained 72 h after transfection for RNA extraction. Knockdown efficiency was measured by qPCR.

### shRNA lentivirus production and sh*METTL3* stable cell line generation

To produce lentiviruses for generating METTL3 knockdown stable lines, HEK293T cells were seeded at 500 K cells per well in antibiotic-free growth media (DMEM medium + 10% heat-inactivated (HI) FBS) in 6-well tissue culture plates. After 24 h, the cells were transfected with psPAX2, pMD2.G and pLKO.1 sh*METTL3* plasmids (500 ng, 50 ng and 500 ng, respectively per well) with TransIT-LT1 transfection reagent (3 μl per well). At 18 h after transfection, media were changed to high-FBS media (DMEM + 30% HI FBS). Lentiviruses were harvested twice at 48 h and 72 h post transfection and combined. The lentiviruses were concentrated using PEG Virus Precipitation Kit (Abcam) following the manufacturer’s instructions.

For lentiviral transduction, 400,000 HEK293T cells were seeded in each well of a 6-well plate. After 24 h, medium was replaced with culture medium supplemented with 10 μl concentrated lentivirus and polybrene at the final concentration of 8 μg ml^−1^. The cells were selected in puromycin-containing medium (1 μg ml^−1^) from 24 h after infection for one week. The knockdown efficiency was measured by qPCR.

### ASO transfection

ASOs were from IDT. The ASO sequences are available in Supplementary Table 6. HEK293T cells were seeded in 48-well plate with the density of 37,500 cells per well one day before transfection. Both gapmer ASOs and steric-blocking ASOs were transfected at a final concentration of 1.5 µM using Lipofectamine RNAiMAX Transfection Reagent, following the manufacturer’s instructions. Cells were obtained 3 days after transfection for downstream analysis.

### RBP overexpression

RBP overexpression was performed by transfection in 24-well plates, with cells seeded at 40–60% confluency, using Lipofectamine 3000 (Thermo Fisher) or jetOPTIMUS (Polyplus, for rescue assay with IGF2BP1 for better co-transfection efficiency) transfection reagents following the manufacturer’s instructions.

### RNA stability assay

RNA stability was measured as described previously^46^ with slight modification. Cells were first transfected with si*RBM15* or non-targeting siRNA (siNT) for 72 h to achieve successful knockdown. The cells were treated with 10 µg ml^−1^ actinomycin D. The cells were collected at time 0 h, 6 h, 12 h, 24 h, 48 h and 72 h time points. RNA was isolated and subjected to RT–qPCR as described above, with equal quantities of RNA from the five time points provided to each reverse transcription reaction. Each RNA was normalized against the 0 h time point to calculate log_2_(fold enrichment).

### circRNA sequencing library preparation

circRNA sequencing libraries were prepared following a homebrewed protocol. In brief, 1 μg of total RNA from overexpression or knockdown samples was subjected to rRNA removal using RiboMinus kit (Thermo Fisher). The rRNA-depleted RNA was poly(A)-tailed with *Escherichia coli* poly(A) polymerase (Thermo Fisher) and digested with 10 U RNase R (Lucigen) at 37 °C for 30 min. Reverse transcription was carried out with random hexamer with PCR handle 1. First-strand cDNA was poly(A)-tailed by terminal deoxynucleotidyl transferase (Thermo Fisher), followed by second-strand synthesis with NEBNext Hifi 2x PCR master mix (NEB) using oligo-dT with PCR handle 2. The double-stranded cDNA was pre-amplified with NEBNext Hifi 2x PCR master mix with PCR handles 1 and 2. Pre-amplified cDNA was then size selected by 0.5x Ampure XP beads (Beckman Coulter) cleanup and subjected to Nextera XT DNA library preparation (Illumina), following the manufacturer’s instructions.

### circRNA sequencing analysis

Reads were processed with ciri quant software^67^. First, reads were adapter-trimmed with skewer with parameter ‘-z -r 0.2 -d 0.2 -q 13 -l 20’, and then we used ciri quant software with default parameters without RNase R correction. The output quantifies the number of back- and forward-splice junction (BSJ and FSJ) reads detected per circRNA, as well as genomic annotations forming the circRNA (exon, intron, antisense, intergenic or unknown).

To quantify expression of each circRNA, we calculated CPM per each BSJ:

$${\mathrm{CP}}\mathrm{{M}}_{i}=1{0}^{6}\times {\rm{BSJ}}_{i}/{\mathrm{total}\; \mathrm{sequencing}\; \mathrm{depth}}$$

We performed the differential expression analysis following ciri quant instruction, ‘Study without biological replicates’ section for the knockdown and overexpression circRNA sequencing screen; and ‘Study with biological replicates’ section for RBM15 overexpression and knockdown in N2A cells.

### rRNA-depleted total RNA-seq and analysis

rRNA-depleted total RNA libraries were prepared with Illumina Stranded Total RNA Prep with Ribo-Zero Plus following the manufacturers’ instruction.

Reads were processed using NF-core RNAseq (available at https://github.com/nf-core/rnaseq) version 3.13.2 (ref. ^68^). In brief, reads were trimmed using trimmomatic (cutadapt v3.4) and mapped using STAR (v2.7.9a). Mapped reads were quantified using Salmon (v1.10.1). Differential expression and splicing analysis compared triplicates of siRNA knockdown of RBM15 against a non-targeting control (HEK_siRBM15/HEK_siNT) as well as triplicates of RBM15 overexpression against a Flag control (RBM15 OE/Flag). For these analyses, DESeq2^69^ (v1.38.3) and rMATS^70^ (v4.3) were used.

### Ribosome profiling and RNA-seq libraries

A replicate of HEK293T was harvested from 10 cm plates for ribosome profiling libraries as previously described^71^. In brief, the cells were treated with 100 μg ml^−1^ cycloheximide for 1 min at 37 °C followed by washing with ice-cold PBS (Corning, 21-040-CV) supplemented with 100 μg ml^−1^ cycloheximide. The cells were lysed with lysis buffer (12.5 mM Tris, pH 7.0; 7.5 mM Tris, pH 8.0; 150 mM NaCl; 5 mM MgCl_2_; 100 μg ml^−1^ cycloheximide; 1 mM DTT; 1% (v/v) Triton X-100; and 20 U ml^−1^ DNase) and centrifuged at 16,000*g* for 10 min at 4 °C. To pellet the ribosomes, the lysate was treated with 250 U RNase I at 25 °C for 45 min, followed by 200 U SUPERase·In RNase Inhibitor (Thermo, AM2694) for quenching, and loaded on sucrose cushion (34% sucrose, 20 mM Tris pH 7.5, 150 mM NaCl, 5 mM MgCl_2_, 1 mM DTT and 100 μg ml^−1^ cycloheximide), followed by 1 h at 100,000 r.p.m. in a TLA-110 rotor (Beckman) at 4 °C. The polysome pellet was resuspended in 1 ml TRIzol (Invitrogen, 15596-018), and RNA was extracted by chloroform-based separation according to manufacturer instructions. The RNA was run on 15% TBE-UREA gel, and 28–34 mRNA fragments were extracted followed by ribosome profiling library construction as described. Corresponding samples were grown in parallel for RNA-seq in six wells as described^72^. In brief, cells were collected with TRI reagent and RNA was extracted using chloroform-based separation. The mRNA was enriched with poly(A) selection (Dynabeads mRNA DIRECT Purification Kit, Invitrogen, 61012), followed by DNA degradation, 3′ mRNA resolving and phosphorylation (DNase I (NEB, M0303S) and 3′ dephosphorylation using FastAP Thermosensitive Alkaline Phosphatase (Thermo, EF0651) and T4 polynucleotide kinase (NEB, M0201S)). RNA ligation with 3′ adapter was carried out using T4 ligase, and reverse transcription with SuperScript III First-Strand Synthesis SuperMix (Invitrogen, 18080400) was carried out for first-strand cDNA synthesis. cDNA products were ligated to second adapter using T4 ligase and amplified by PCR for final library products.

### Computational analysis for ribosome profiling and paired RNA-seq

Sequencing reads were aligned as previously described^72^. In brief, linker (CTGTAGGCACCATCAAT) and poly(A) sequences were removed, and the remaining reads were aligned to rRNA. Then, unaligned rRNA reads were used for alignment to GRCh38 human sequence using Bowtie v1.1.229 (ref. ^73^) with a maximum of two mismatches per read. Unaligned reads to the genome were used for alignment to the sequences that spanned splice junctions. In ribosome profiling analysis, the ribosomal P-site was calculated by using the 5′ position of the aligned reads of the ORFs, with +12 offset for reads that were 28–29 bases and +13 offset for reads that were 30–33 bases^74,75^.

### AP-MS

HEK293T cells were lysed and affinity purified using 10 µg per sample of anti-RBM15 antibody (Bethyl). In brief, the cell lysates with antibody were incubated with magnetic beads overnight in the cold room. Then, 5 µl of 10 mg ml^−1^ RNase A was added to ribonuclease-positive conditions at this step. Supernatants were removed, and beads were washed with NP-40 buffer, twice in buffer 2 (50 mM Tris (pH 7.5), 150 mM NaCl, 10 mM MgCl_2_, 0.05% NP-40 and 5% glycerol) and twice in buffer 3 (50 mM Tris (pH 7.5), 150 mM NaCl, 10 mM MgCl_2_ and 5% glycerol). After the last wash, the wash buffer was spun down and aspirated completely, and the beads were resuspended in 80 μl of trypsin buffer (2 M urea, 50 mM Tris (pH 7.5) and 5 μg ml^−1^ trypsin) to digest the bound proteins at 37 °C for 1 h with agitation. The beads were centrifuged at 100*g* for 30 s, and the partially digested proteins (the supernatant) were collected. The beads were then washed twice with 60 μl of urea buffer (2 M urea and 50 mM Tris (pH 7.5)). The supernatant of both washes was collected and combined with the partially digested proteins (final volume, 200 μl). After brief centrifugation, the combined partially digested proteins were cleared from residual beads. Then, 80 µl of these partially digested proteins was used; disulfide bonds were reduced with 5 mM DTT; and cysteines were subsequently alkylated with 10 mM iodoacetamide. Samples were further digested by adding 0.5 μg of sequencing-grade modified trypsin (Promega) at 25 °C. After 16 h of digestion, samples were acidified with 1% formic acid (final concentration). Tryptic peptides were desalted on C18 StageTips according to ‘Protocol for micro-purification enrichment pre-fractionation and storage of peptides for proteomics using StageTips’^76^, and evaporated to dryness in a vacuum concentrator and reconstituted in 15 μl of 3% acetonitrile, 2% formic acid for liquid chromatography with tandem mass spectrometry (LC–MS/MS).

LC–MS/MS analysis was performed on a Q Exactive HF. Five microliters of total peptides was analyzed on a Waters M-Class UPLC using a 15 cm IonOpticks column (1.7 µm, 120 Å and 75 µm × 15 cm) coupled to a benchtop Thermo Fisher Scientific Orbitrap Q Exactive HF mass spectrometer. Peptides were separated at a flow rate of 400 nl min^−1^ with a 90 min gradient, including sample loading and column equilibration times. Data were acquired in data-dependent mode. MS1 spectra were measured with a resolution of 120,000, an automatic gain control (ACG) target of 3 × 10^6^ and a mass range from 300 *m*/*z* to 1,800 *m*/*z*; MS2 spectra were measured at a resolution of 15,000, an AGC target of 1 × 10^5^, a TopN of 12, an isolation window of 1.6 *m*/*z* and a mass range from 200 *m*/*z* to 2,000 *m*/*z*.

### AP-MS data analysis

Proteomics raw data were analyzed by MaxQuant v2.0.3.0 (ref. ^77^) using a UniProt database (*Homo sapiens*, UP000005640), and MS/MS searches were performed under default settings with label-free quantification (LFQ) and match between the runs. Data were further analyzed in R v3.6.3. Contaminants and proteins only identified by site or reverse were removed, LFQ intensity values were transformed to parts per million, a pseudocount of 1 was added, and then the values were log_2_ transformed. Proteins with a mean MS/MS count value for each immunoprecipitation condition below 5 were removed from subsequent analysis and missing values were imputed with values from the bottom of the signal distribution for measured protein intensities. Interacting proteins were identified as those that passed a log_2_FC sample immunoprecipitation over control immunoprecipitation cutoff of 1 and a *P* value of 0.05. Sample and control immunoprecipitations were matched by RNase treatment.

### eCLIP and eCLIP data analysis

eCLIP was performed as previously described^78^.

Computational analysis of eCLIP data was performed using the default settings of Skipper^79^ available on GitHub (https://github.com/YeoLab/skipper). Reads were mapped to human genome assembly GRCh38.

### RIP and RIP–circRNA sequencing analysis

RIP experiments were performed as previously described^80^ with slight modifications. In brief for each replicate, around 20 million HEK293T cells were harvested, washed with cold PBS and lysed on ice with lysis buffer (150 mM NaCl, 50 mM Tris-HCl, pH 8, 5 mM MgCl_2_, 1 mM CaCl_2_, 0.5 mM EDTA, 0.5% NP-40, 1:200 proteinase inhibitor and 1:500 RNaseOUT). The lysates were centrifuged at 18,000*g* for 10 min at 4 °C to remove cell debris. The supernatant was first incubated with lysis buffer-washed Protein A beads at 4 °C for 30 min to preclear non-specific binding of the beads. Meanwhile, additional Protein A Dynabeads (Invitrogen, 10002D) were washed and incubated with respective antibodies (anti-LARP4B, anti-RBM15 and control rabbit IgG) in lysis buffer at room temperature for 15 min. Protein A beads coated with antibodies were then washed twice with lysis buffer. Then, 30 µl of the lysate supernatant was kept as input, while 450 µl of the lysate supernatant was added to either anti-LARP4B/anti-RBM15 coated or control IgG coated Protein A beads and incubated at 4 °C overnight. After the incubation, the beads were washed three times with lysis buffer. The immunoprecipitated RNA was released by proteinase K digestion, and both the immunoprecipitatedI and input RNA were cleaned up by the RNA Clean & Concentrator-5 kit following manufacturers’ instructions. The resulting RNA was subjected to circRNA library preparation or reverse transcription followed by qPCR quantifications.

The statistical procedure is similar to that presented in Skipper^79^. Let there be two libraries: foreground library (immunoprecipitatedI), and background library (Input). We use beta-binomial distribution as the null distribution to model the BSJ counts between foreground and background. The overdispersion term is obtained by pairwise comparing the replicates. The probability of success is set to the fraction of total BSJs in the foreground library.

### Integrated analysis of eCLIP and circRNA sequencing

RBM15 positively regulated circRNAs were converted to bed format using bedtools^81^. eCLIP bed files of reproducible binding sites were generated as Skipper output. The overlaps between circRNAs and eCLIP bed files were identified by the intersect function from bedtools. Odds ratios were calculated and Fisher’s exact test or chi-square test was used to calculate *P* values.

### Metadensity plot

To visualize RBM15 eCLIP signals around circRNAs, we utilize Metadensity^82^, a package that aggregates eCLIP raw signals and plots against selected genomic annotations. circRNAs are stratified into three groups, upregulated upon knockdown (*n* = 614), downregulated upon knockdown (*n* = 4) and those without change. We randomly sampled 500 circRNAs without change as comparison. Each circular BSJ was built into the gene annotation, and ENCODE HepG2 eCLIP signals were plotted around the BSJ-forming interval. Specifically, we utilize the ‘relative information’ method to quantify the difference between immunoprecipitated and size-matched input.

### *Drosophila* stocks and culture

*UAS-myc-nito* was a gift from T. Reis^83^. To generate the mCherry-RNAi transgene, we cloned a short hairpin into P[WALIUM20] (DGRC_1472) yielding P[WALIUM20-mCherry]. The hairpin was made using the following oligonucleotides. Forward: ctagcagtCGAGTTCATCTACAAGGTGAAtagttatattcaagcataTTCACCTTGTAGATGAACTCGgcg,

Reverse: aattcgcCGAGTTCATCTACAAGGTGAAtatgcttgaatataactaTTCACCTTGTAGATGAACTCGactg.

This was injected into *y*^*1*^ *w*^*1118*^*; attP2[nos-Cas9]/TM6C, Sb Tb* to obtain transformants (BestGene, Inc.). Other stocks were obtained from the Bloomington Drosophila Stock Center: *elav*^*C155*^*-Gal4* (BL-458), *nito-RNAi* (BL-56851) and *UAS-DsRed* (BL-6282). Flies were raised on standard cornmeal-based food medium containing 1.25% w/v agar, 10.5% w/v dextrose, 10.5% w/v maize and 2.1% w/v yeast at 60% relative humidity.

### *Drosophila* immunostaining

We performed immunostaining as previously described^84^, by fixing dissected tissues in PBS containing 4% formaldehyde and incubating with the following primary antibodies: mouse anti-Fl(2)d (1:1,000 DSHB 9G2), and rabbit anti-Nito (1:2,000, gift from N. Perrimon^85^). Alexa Fluor-488, -568 and -647 secondary antibodies were from Invitrogen and used at 1:1,000. Tissues were mounted in Vectashield mounting buffer with DAPI (Vector Laboratories). Images were captured with a Leica STELLARIS confocal microscope; endogenous red fluorescent protein (RFP) signals were monitored.

### *Drosophila* sample collection and RNA-seq

#### *nito* loss-of-function studies

Adult flies of the specified genotypes (control, *elav-Gal4/+;; UAS-mCherry-RNAi/+*; experimental, *elav-Gal4/+; UAS-nito-RNAi/+*) were aged to 1 to 2 days at 25 °C. We selected this as the latest temporal window we could analyze, as most *nito*-knockdown animals died after 2 days of adulthood. We dissected 12–15 female heads to prepare RNA.

#### *nito* gain-of-function studies

Third instar larvae of the specified genotypes (control, *elav-Gal4;; UAS-DsRed/+;* experimental, *elav-Gal4; UAS-myc-nito/+*) were collected at 18 °C. We selected this as the latest temporal window we could analyze, since misexpression of Nito was lethal before pupation, even when lowering Gal4 activity by culturing at 18 °C. We dissected the central nervous system from 6 to 7 larvae (mixed sex) for RNA isolation.

In both genetic manipulations, we extracted total RNA using TRIzol (Invitrogen). Sequencing libraries were prepared using the QIAseq FastSelect –rRNA Fly Kit (Qiagen) for rRNA depletion, followed by the TruSeq stranded mRNA Kit (Illumina) using the manufacturer’s protocols.

Sequencing was performed on a NovaSeq X Plus in paired end read mode. Reads were processed with ciri quant software^67^ (mapped to BDGP Release 6/dm6). We performed the differential expression analysis following ciri quant instruction, ‘Study with biological replicates’ section, which has accounted for the expression changes of the linear cognates^67^.

### Reporting summary

Further information on research design is available in the Nature Portfolio Reporting Summary linked to this article.

## Online content

Any methods, additional references, Nature Portfolio reporting summaries, source data, extended data, supplementary information, acknowledgements, peer review information; details of author contributions and competing interests; and statements of data and code availability are available at https://doi.org/10.1038/s41594-026-01911-7.

## Supplementary information

Reporting Summary

Peer Review File

Supplementary Table 1 Summary of RBP tethering screen results.

Supplementary Table 2 Summary of secondary screen and exocircFirefly screen results.

Supplementary Table 3 List of RBP positively regulated and negatively regulated circRNAs.

Supplementary Table 4 RBM15 AP-MS results.

Supplementary Table 5 List of conserved circRNAs RBM15 positively regulated in N2A cells.

Supplementary Table 6 List of primers, ASOs and antibodies.

Supplementary Table 7 Plasmid maps.

## Source data

Source Data Raw western images for Figs. 3a,e,f, 4c and 6b–d.

Source Data Source data for Figs. 1–7 and Extended Data Figs. 1–9.

## Data Availability

All sequencing data generated in this study can be found in the Gene Expression Omnibus (GEO) under accession GSE314435. Source data are provided with this paper.

## Code availability

The code for the custom software used in this study can be found via GitHub at https://github.com/YeoLab, including Skipper and Metadensity. Other computational analyses were described in the Methods.
