# Supplementary material for: Large-scale RNA-binding protein tethering screen discovers modulators of circular RNA metabolism
Source: Nat Struct Mol Biol. 2026 Nov 4;33(11):1676–89. doi: 10.1038/s41594-026-01911-7 (PMC13759317; doi:10.1038/s41594-026-01911-7)
Supplement: Supplementary file 1 [file 41594_2026_1911_MOESM10_ESM.pdf]

Fig 3e

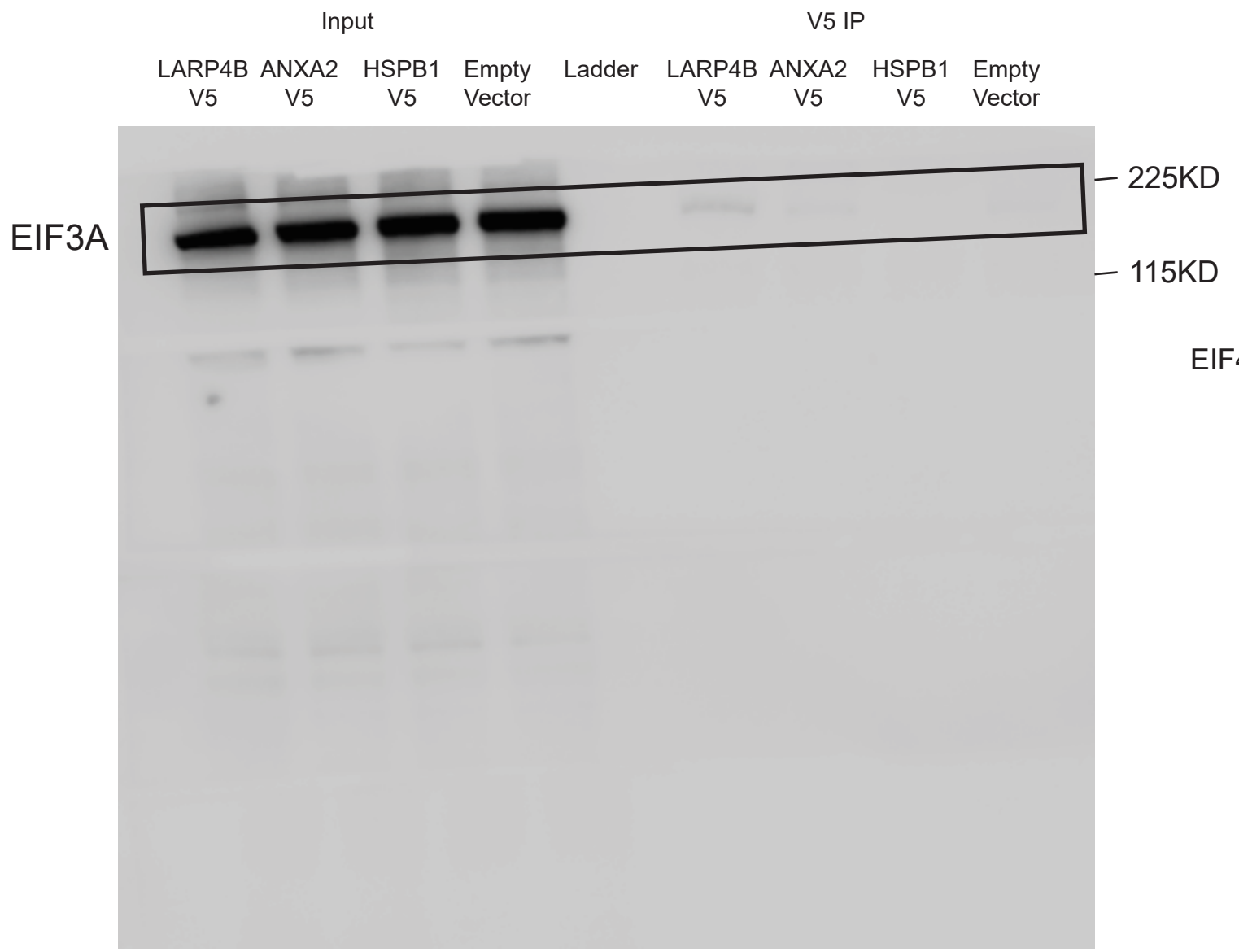
Input
V5 IP
LARP4B V5
ANXA2 V5
HSPB1 V5
Empty Vector
Ladder
LARP4B V5
ANXA2 V5
HSPB1 V5
Empty Vector
EIF3A
225KD
115KD

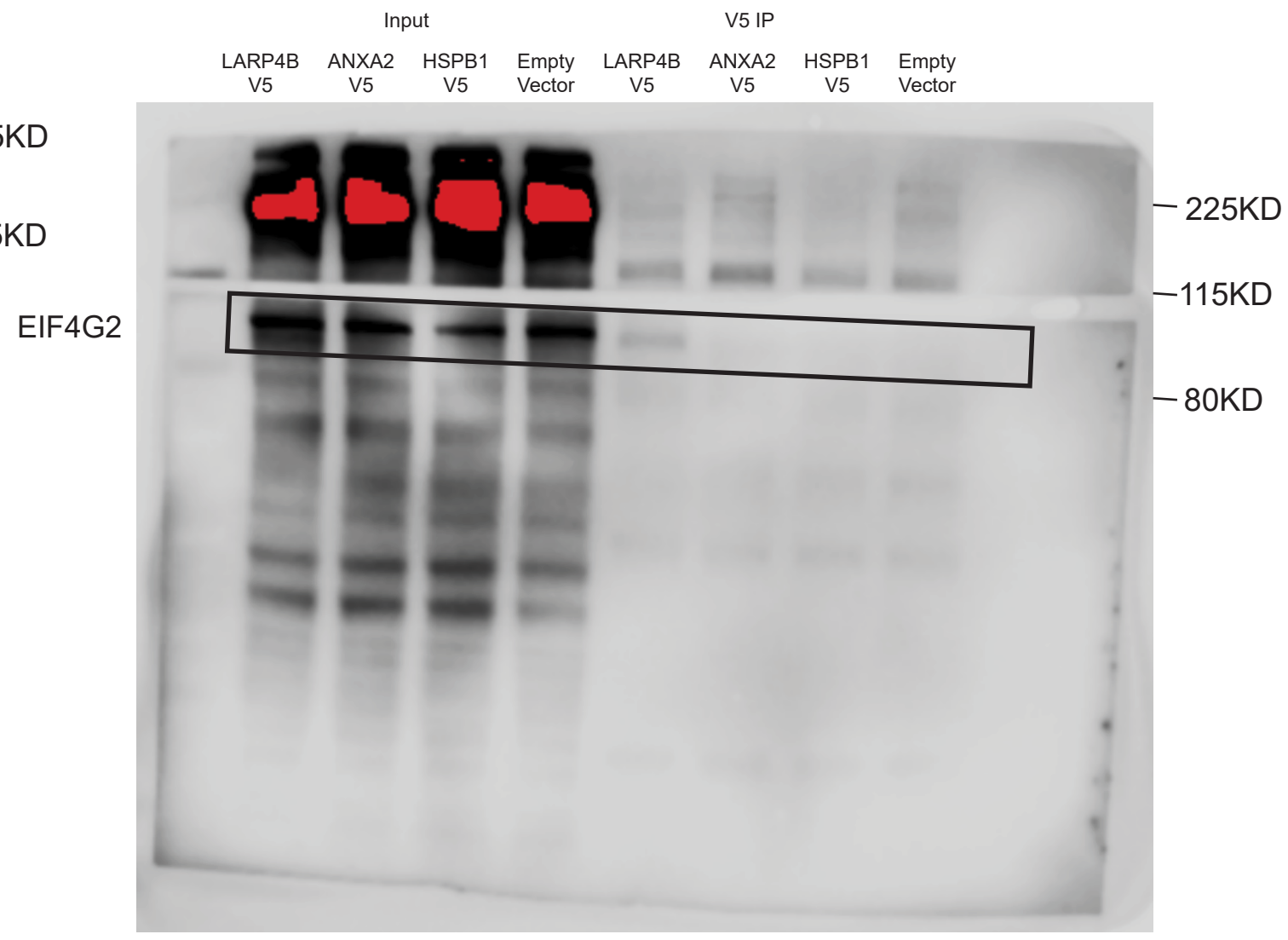
Input
V5 IP
LARP4B V5
ANXA2 V5
HSPB1 V5
Empty Vector
LARP4B V5
ANXA2 V5
HSPB1 V5
Empty Vector
EIF4G2
225KD
115KD
80KD

## Extended Data Fig 3a

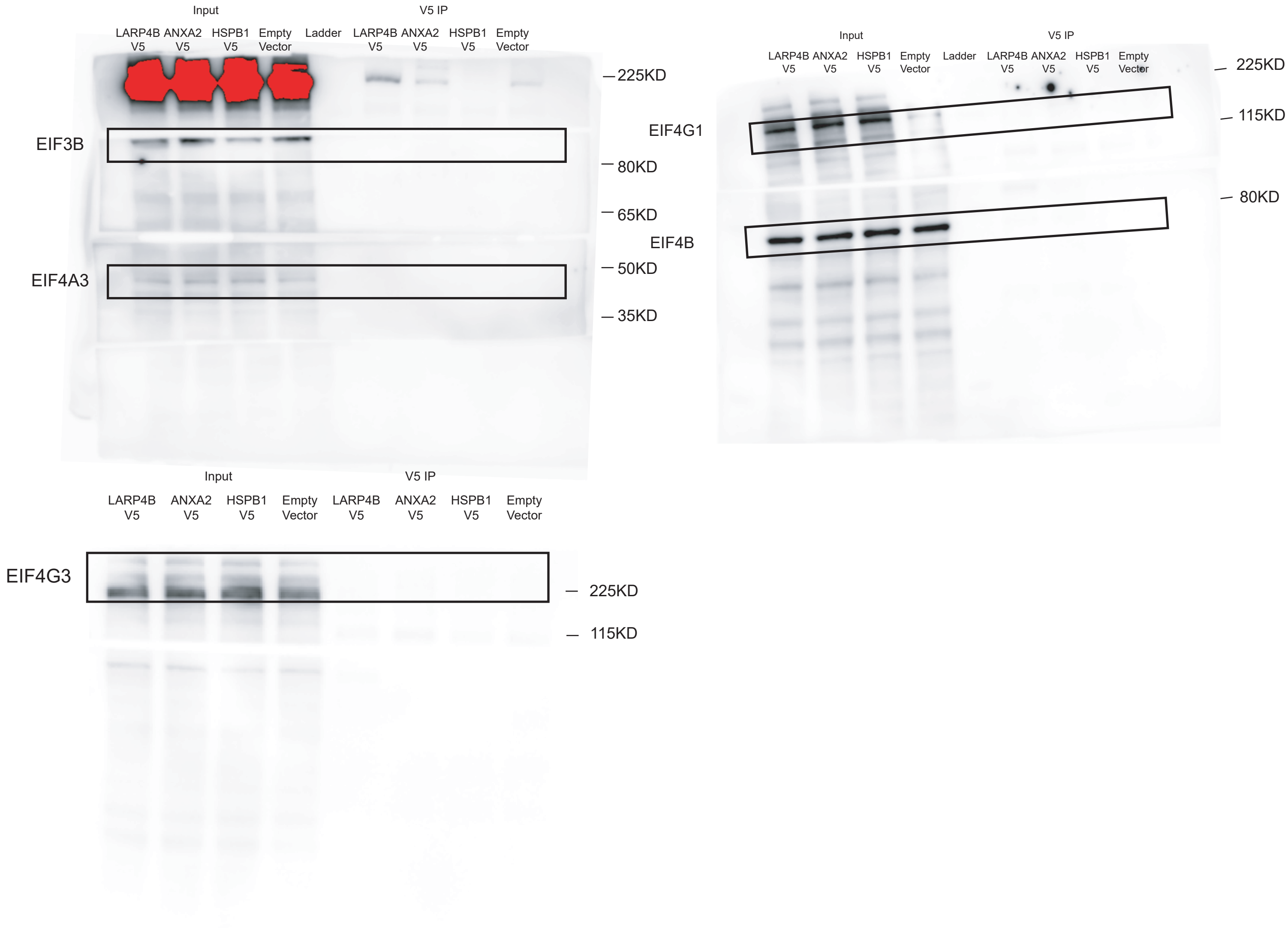


## Extended Data Fig 3f

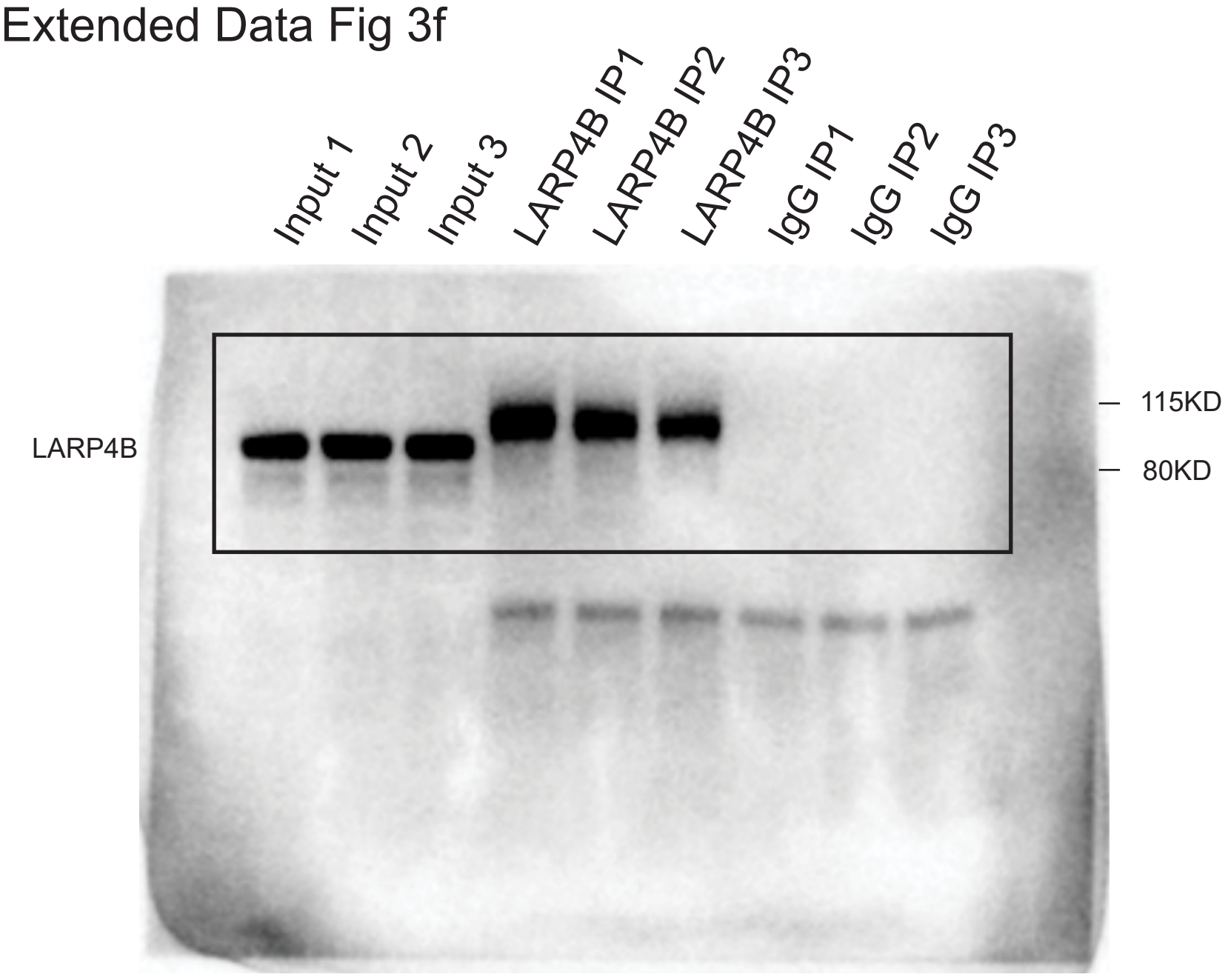

Extended Data Fig 4c

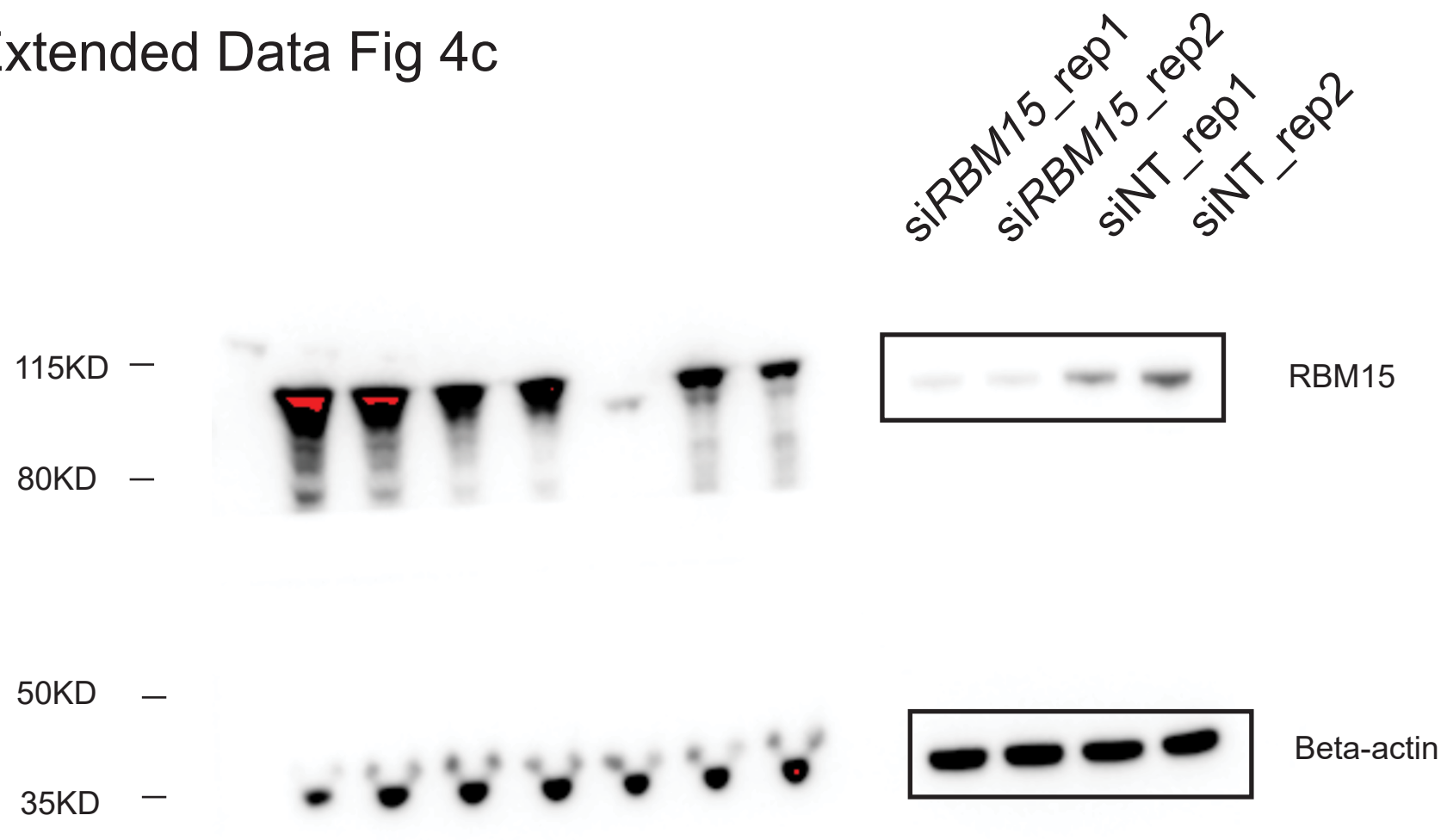


Reference gene from the same blot but cut for different primary antibody incubation.

Extended Data Fig 6d

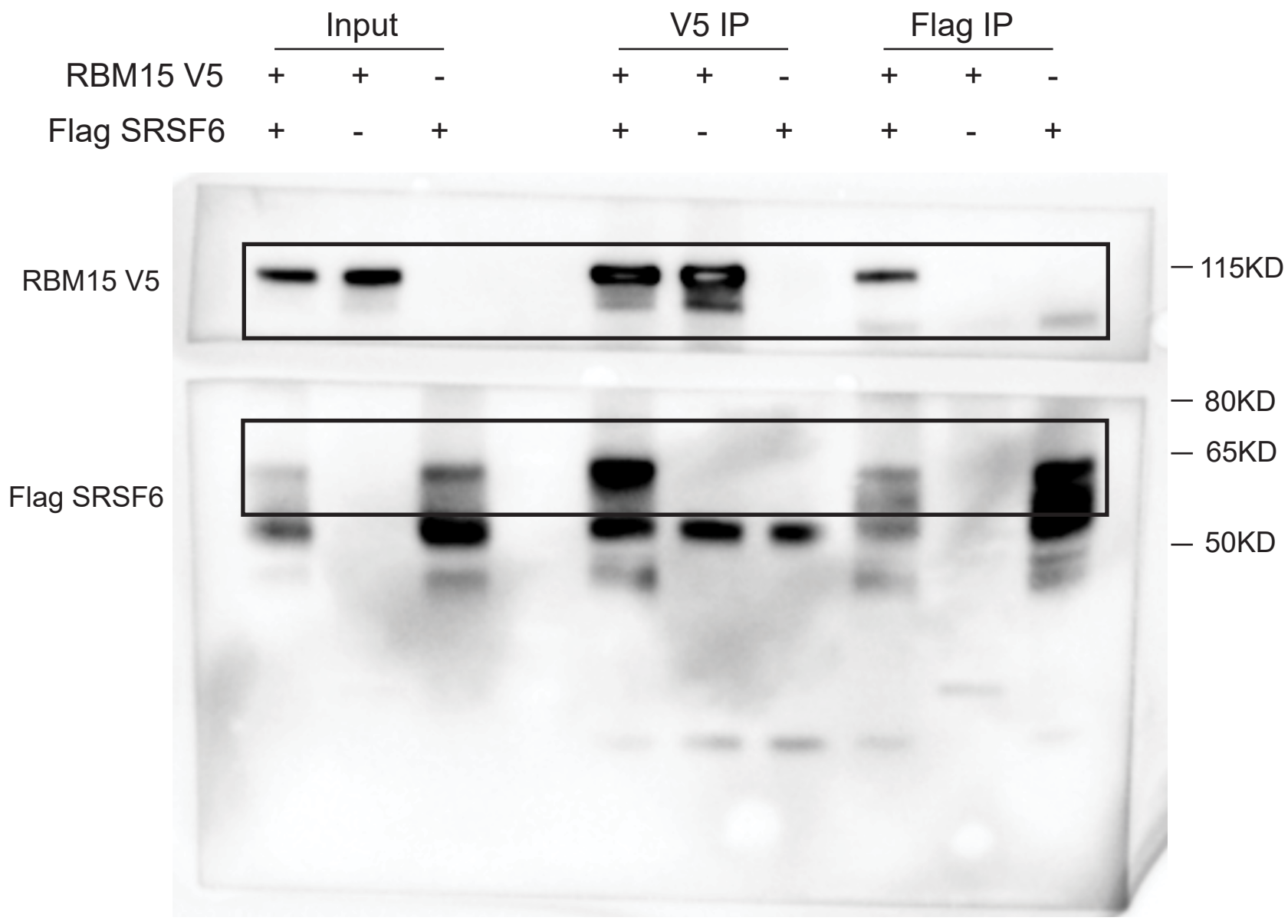

Fig 6b,c

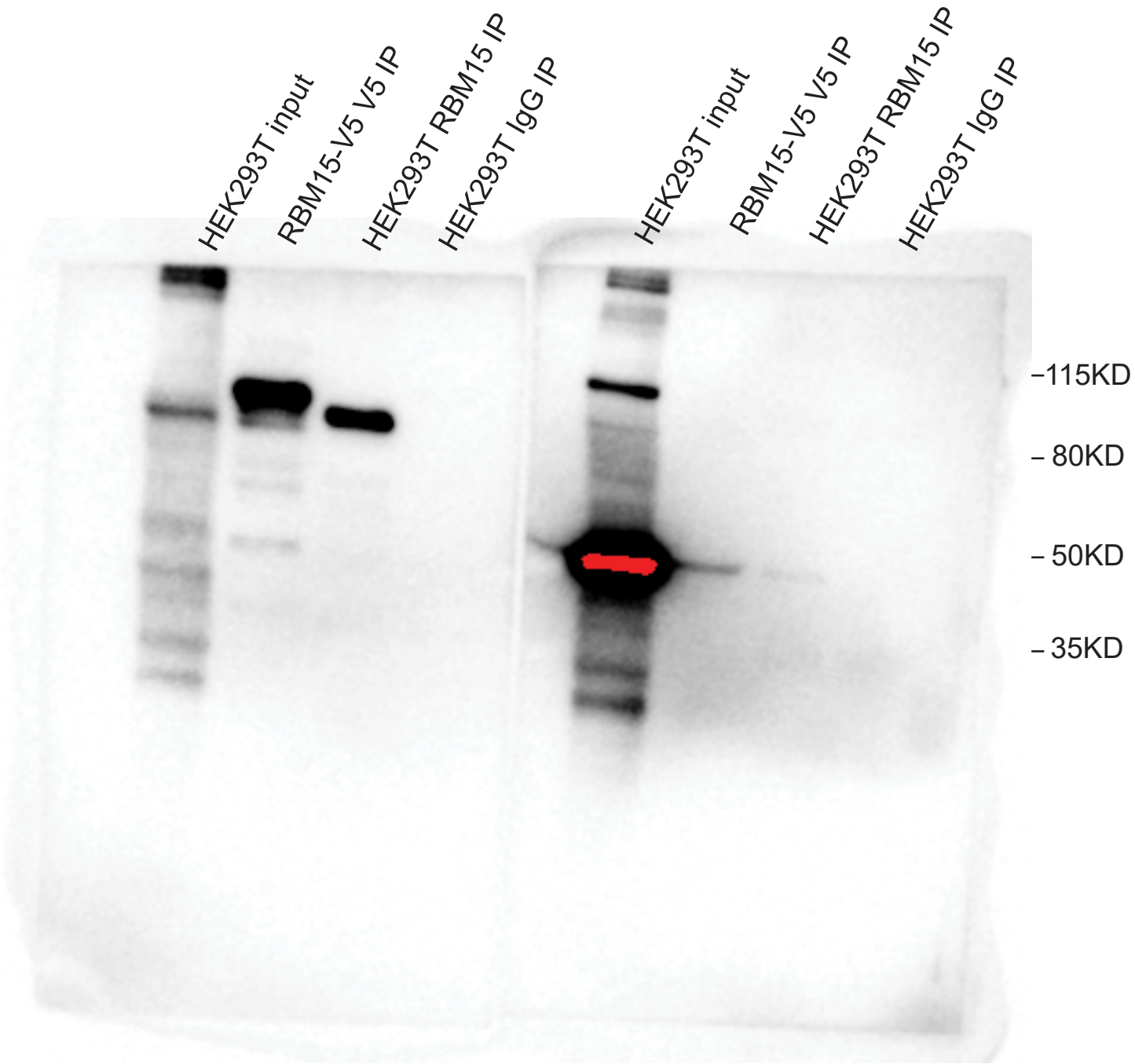
HEK293T input
RBM15-V5 V5 IP
HEK293T RBM15 IP
HEK293T IgG IP
HEK293T input
RBM15-V5 V5 IP
HEK293T RBM15 IP
HEK293T IgG IP
115KD
80KD
50KD
35KD
