# Supplementary material for: Large-scale RNA-binding protein tethering screen discovers modulators of circular RNA metabolism
Source: Nat Struct Mol Biol. 2026 Nov 4;33(11):1676–89. doi: 10.1038/s41594-026-01911-7 (PMC13759317; doi:10.1038/s41594-026-01911-7)
Supplement: Supplementary file 3 [file 41594_2026_1911_MOESM2_ESM.pdf]

nature portfolio

Peer Review File

Large-scale RNA-binding protein tethering screen discovers modulators of circular RNA metabolism

Corresponding Author: Professor Gene Yeo

This manuscript has been previously reviewed at another journal that is not operating a transparent peer review scheme. The manuscript was considered suitable for publication without further review at Nature Structural & Molecular Biology.

Version 0:

Decision Letter:

Our ref: NSMB-A52790-T

14th Jul 2026

Dear Dr. Yeo,

Thank you for submitting your revised manuscript "Large-scale RNA-binding protein tethering screen discovers modulators of circular RNA metabolism" (NSMB-A52790-T). I sincerely apologize for the delay in responding, as we discussed the manuscript file with the editorial team. We find that the manuscript now has improved in revision and we'll be happy in principle to publish it in Nature Structural & Molecular Biology, pending minor revisions to satisfy the referees' final requests and/or to comply with our editorial and formatting guidelines.

We are now performing detailed checks on your paper and will send you a checklist detailing our editorial and formatting requirements in a couple of weeks. Please do not upload the final materials and make any revisions until you receive this additional information from us.

To facilitate our work at this stage, it is important that we have a copy of the main text as a word file. If you could please send along a word version of this file as soon as possible, we would greatly appreciate it; please make sure to copy the NSMB account (cc'ed above).

Thank you again for your interest in Nature Structural & Molecular Biology Please do not hesitate to contact me if you have any questions.

Sincerely,
Sara

Sara Osman, Ph.D.
Senior Editor
Nature Structural & Molecular Biology

INTERNAL USE ONLY AIP modules required: ["Clinical","Life Science","Chemistry"]

Open Access This Peer Review File is licensed under a Creative Commons Attribution 4.0 International License, which permits use, sharing, adaptation, distribution and reproduction in any medium or format, as long as you give appropriate credit to the original author(s) and the source, provide a link to the Creative Commons license, and indicate if changes were made.
In cases where reviewers are anonymous, credit should be given to 'Anonymous Referee' and the source.
The images or other third party material in this Peer Review File are included in the article's Creative Commons license, unless indicated otherwise in a credit line to the material. If material is not included in the article's Creative Commons license and your intended use is not permitted by statutory regulation or exceeds the permitted use, you will need to obtain permission directly from the copyright holder.
To view a copy of this license, visit https://creativecommons.org/licenses/by/4.0/
